# Supplementary figures and images for: Finding, visualizing, and quantifying latent structure across diverse animal vocal repertoires
Source: PLoS Comput Biol. 2020 Oct 15;16(10):e1008228. doi: 10.1371/journal.pcbi.1008228 (PMC7591061; doi:10.1371/journal.pcbi.1008228)

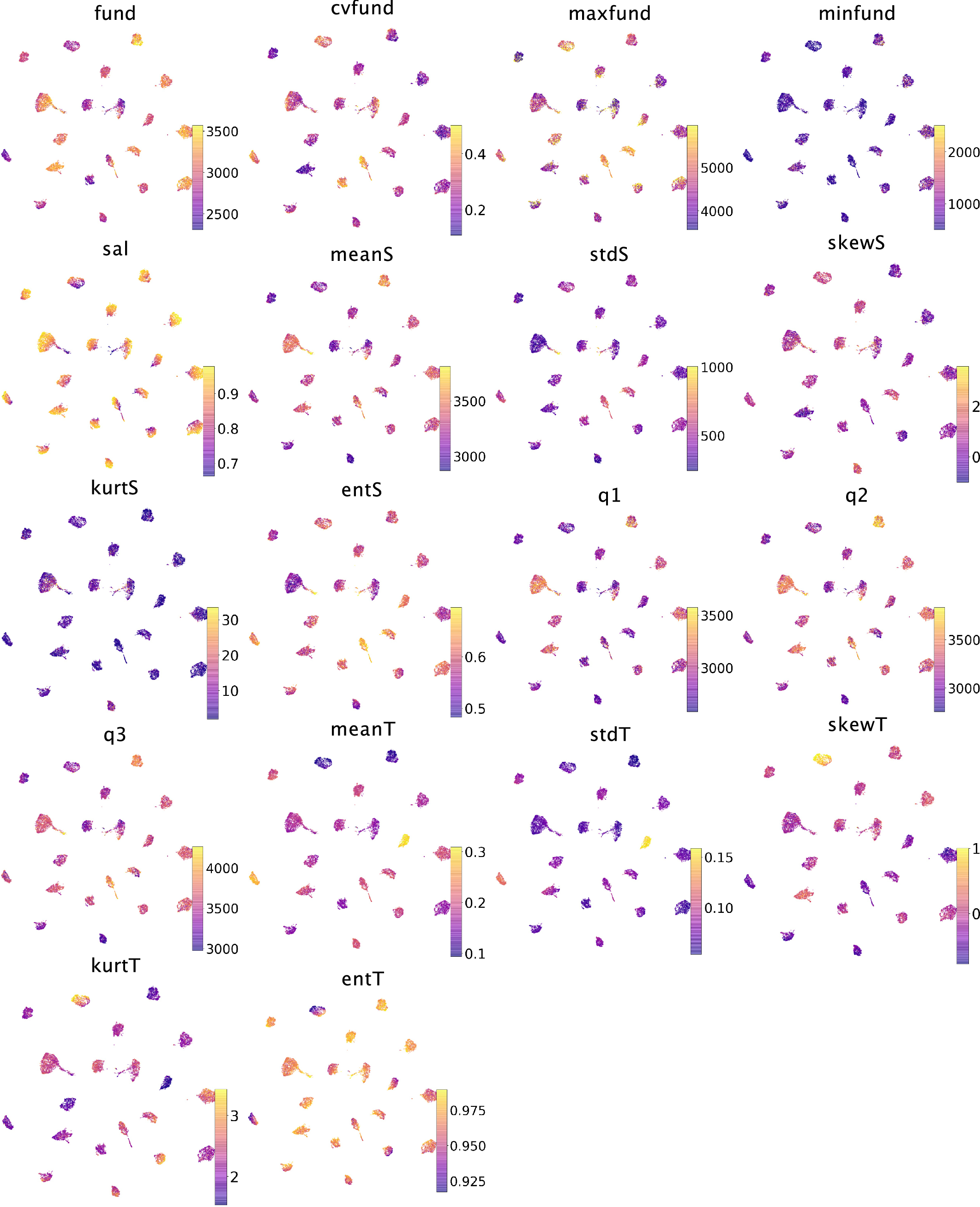

Supplement: S1 Fig — (A) More information regarding each feature can be found in S2 Table and Elie et al. [24, 28]. (TIF) [file pcbi.1008228.s001.tif]

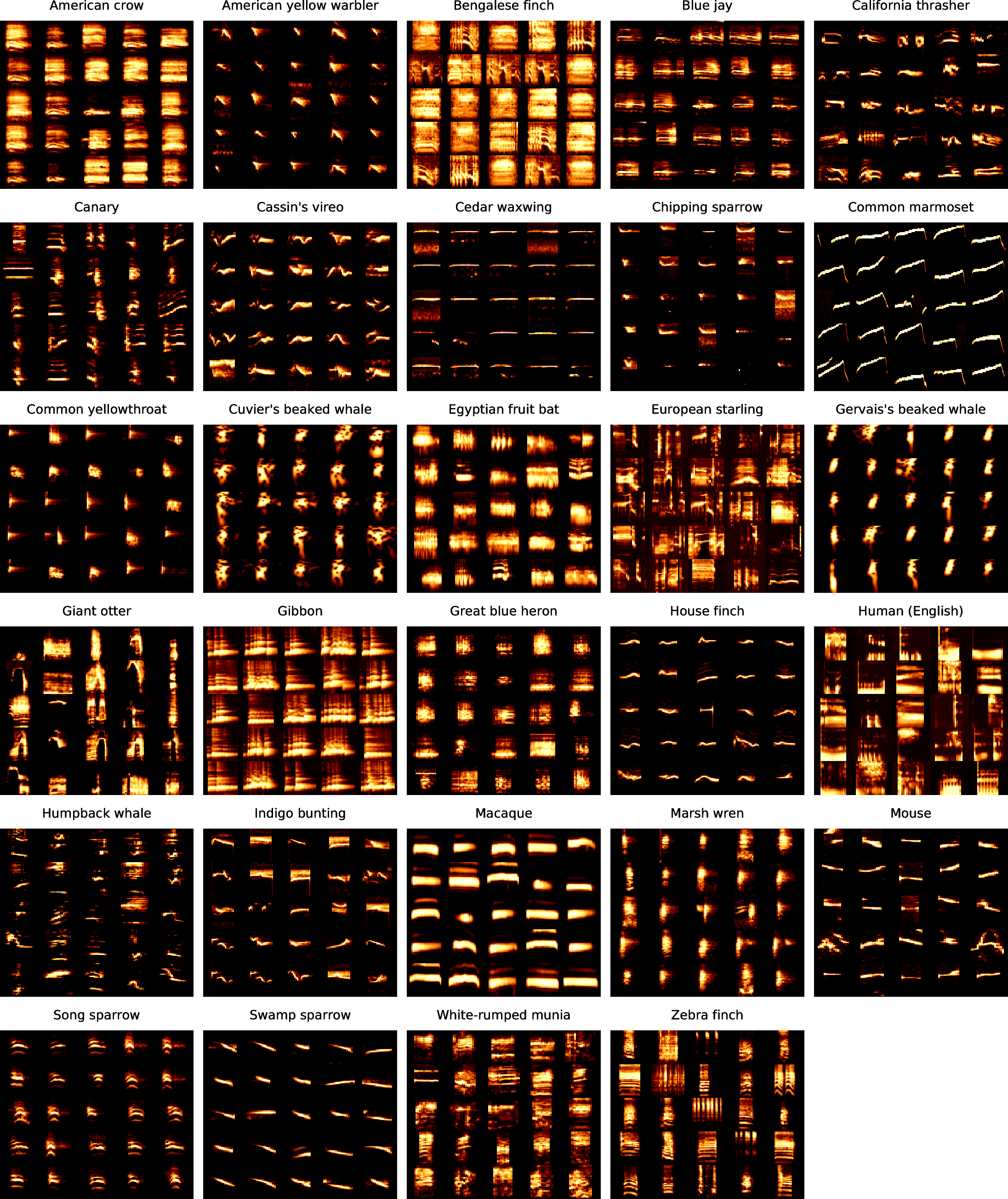

Supplement: S2 Fig — (TIF) [file pcbi.1008228.s002.tif]

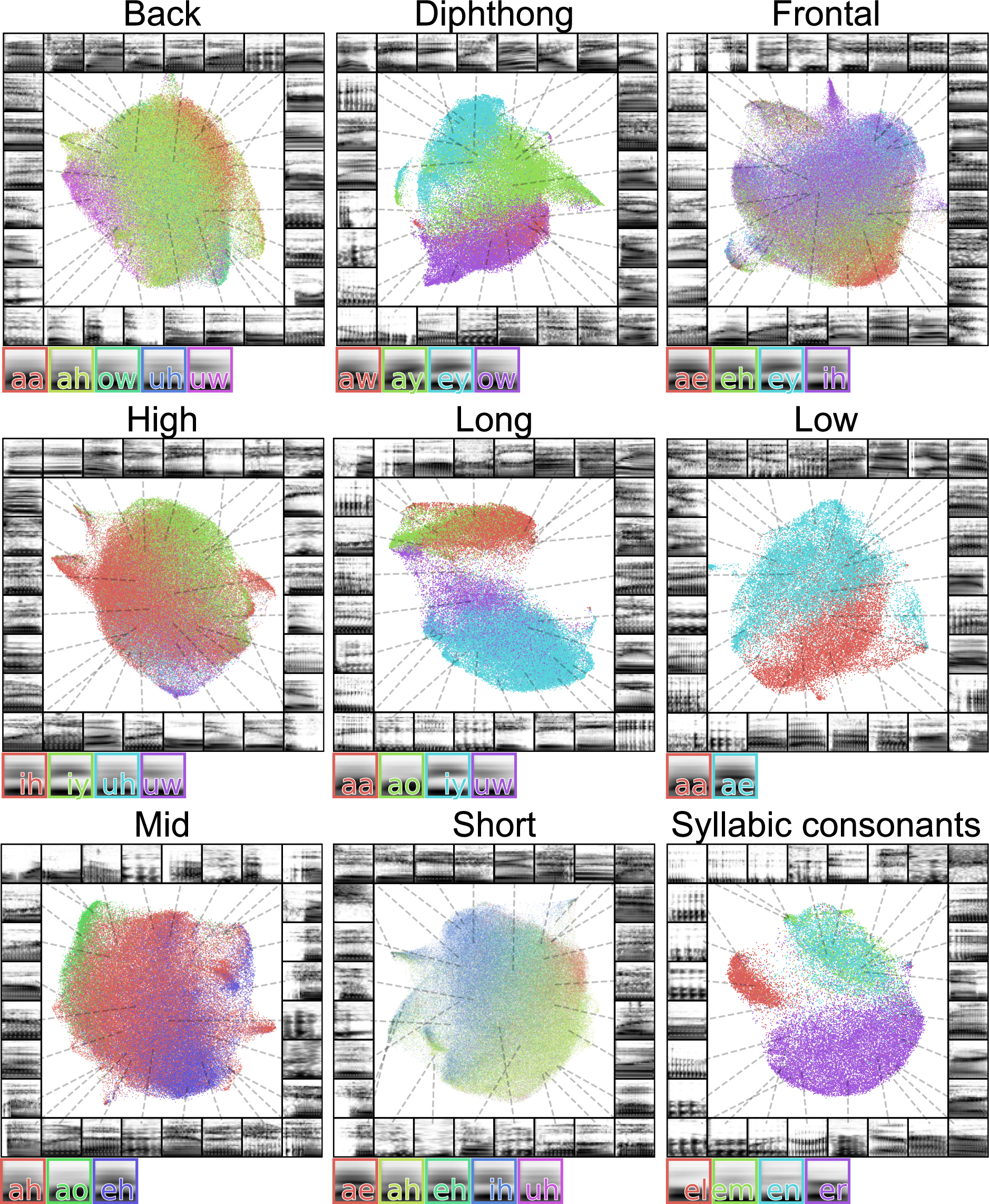

Supplement: S3 Fig — Each plot shows a different set of vowels grouped by phonetic features. The average spectrogram for each vowel is shown to the right of each plot. (TIF) [file pcbi.1008228.s003.tif]

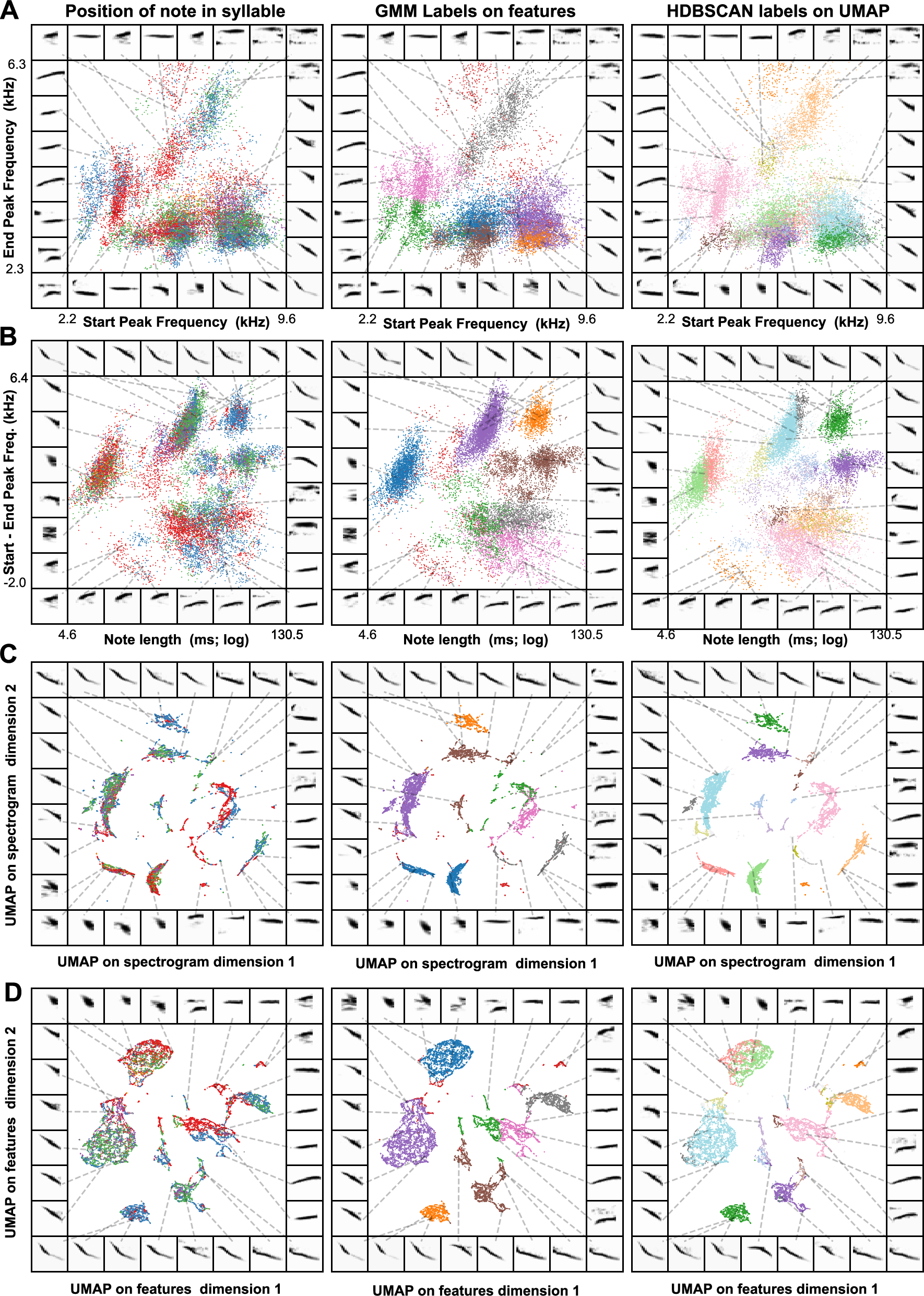

Supplement: S4 Fig — (A) A scatterplot of the start and end peak frequencies of the notes produced by birds recorded in Hudson Valley, NY. The left panel shows notes colored by the position of each note in the syllable (red = first, blue = second, green = third). The center panel shows the sample scatterplot colored by a Gaussian Mixture Model labels (fit to the start and end peak frequencies and the note duration). The right panel shows the scatterplot colored by HDBSCAN labels over a UMAP projection of the spectrograms of notes. (B) The same notes, plotting the change in peak frequency over the note against the note’s duration. (C) The same notes plotted as a UMAP projection over note-spectrograms. (D) The features from (A) and (B) projected together into a 2D UMAP space. (TIF) [file pcbi.1008228.s004.tif]

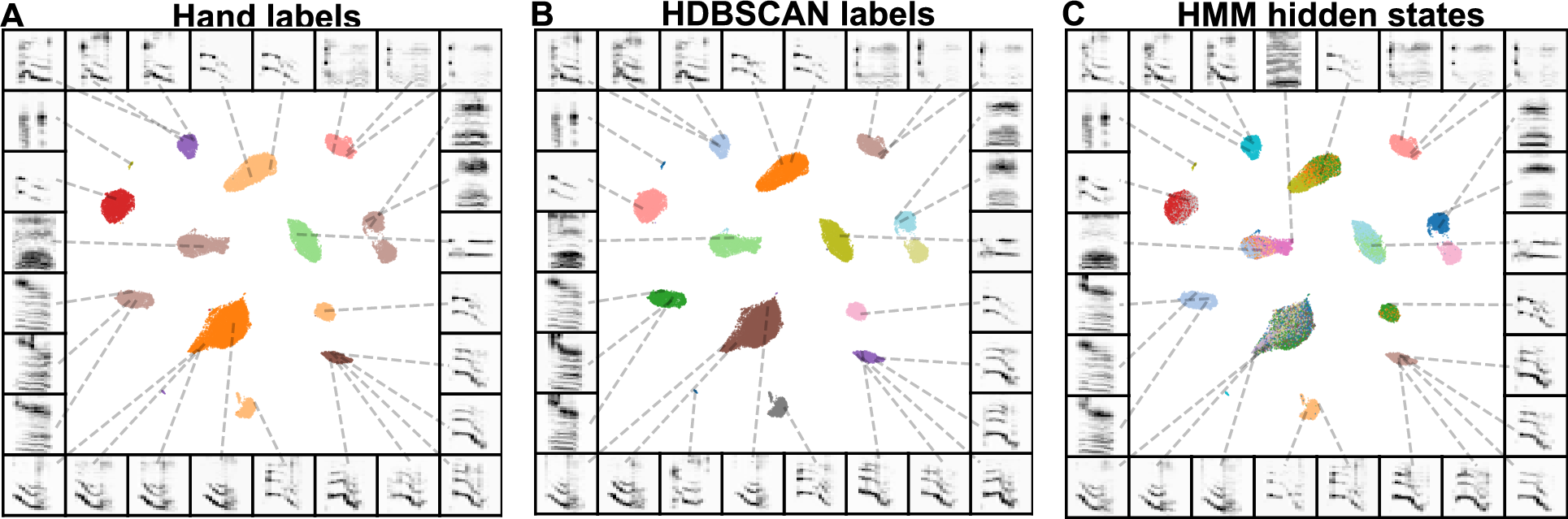

Supplement: S5 Fig — Projections are shown for a single example bird from the Nicholson dataset [63]. UMAP projections are labeled by three labeling schemes: (A) Hand labels, (B) HDBSCAN labels on UMAP, and (C) Trained Hidden Markov Model (HMM) labels. (TIF) [file pcbi.1008228.s005.tif]

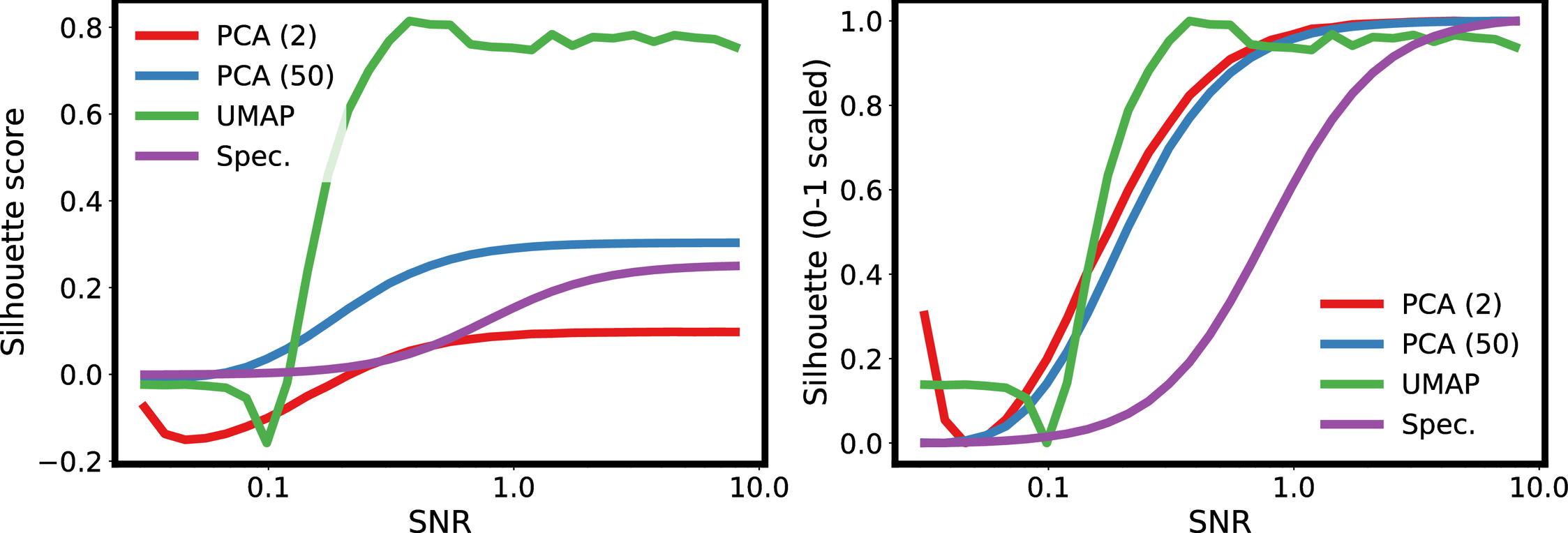

Supplement: S6 Fig — White noise is added to the spectrogram to modulate signal to noise ratio (SNR). The different projections (2-dimensional PCA, 50-dimensional PCA, 2-dimensional UMAP) and the spectrogram are compared on the basis of silhouette score for the labels of each Cassin’s vireo syllable. The left panel shows the silhouette score, and the right panel shows the silhouette score scaled between 0 and 1 to more easily compare change as a function of SNR. (TIF) [file pcbi.1008228.s006.tif]
