# Supplementary material for: Finding, visualizing, and quantifying latent structure across diverse animal vocal repertoires
Source: PLoS Comput Biol. 2020 Oct 15;16(10):e1008228. doi: 10.1371/journal.pcbi.1008228 (PMC7591061; doi:10.1371/journal.pcbi.1008228)
Supplement: S1 Table — (PDF) [file pcbi.1008228.s007.pdf]

Species	# Indv.	# Elements	Median len. (s)	Total length (s)	# Rec.	References
American crow	Unk.	syllables: 252	syllables: 0.37	100.5	252	[50, 107]
Bengalese finch	4	syllables: 215480	syllables: 0.065	40205.6	2663	[63]
Bengalese finch	11	notes: 214915	notes: 0.089	35365.9	2964	[8, 64]
Blue jay	Unk.	syllables: 250	syllables: 0.47	141.2	250	[50, 107]
California thrasher	18	syllables: 15328	syllables: 0.146	19958.9	92	[6, 41]
Canary	5	phrases: 22167<br>syllables: 497338	phrases: 1.319<br>syllables: 0.04	36986.9	2320	[5]
Cassin's vireo	48	syllables: 67316	syllables: 0.332	434782.4	422	[7, 41]
Cedar waxwind	Unk.	syllables: 245	syllables: 0.425	116.0	245	[50, 107]
Chipping sparrow	Unk.	syllables: 252	syllables: 0.09	24.9	252	[50, 107]
Common marmoset	33	calls: 14289	calls: 1.084	76400.7	768	[47]
Common yellowthroat	Unk.	syllables: 255	syllables: 0.1	35.4	255	[50, 107]
Cuvier's beajed whale	Unk.	clicks: 2237	clicks: 0.001	2.3	2237	[51, 106]
Egyptian fruit bat	83	syllables: 423043	syllables: 0.042	166676.8	83823	[103, 104]
European starling	7	syllables: 164230	syllables: 0.577	194529.9	3805	[101]
Gervais's beaked whale	Unk.	clicks: 1936	clicks: 0.001	2.0	1936	[51, 106]
Giant otter	Unk.	syllables: 452	syllables: 0.68	390.4	452	[98]
Gibbon	Unk.	syllables: 10333	syllables: 2.96	230400.0	128	[102]
Great blue heron	Unk.	syllables: 246	syllables: 0.138	44.1	246	[50, 107]
House finch	Unk.	syllables: 248	syllables: 0.093	25.9	248	[50, 107]
Human (English)	40	words: 283721<br>phones: 837896	words: 0.205<br>phones: 0.069	135708.4	254	[96]
Humpback whale	Unk.	syllables: 2006	syllables: 1.65	6730.8	13	[100]
Indigo bunting	Unk.	syllables: 251	syllables: 0.135	36.0	251	[50, 107]
Macaque	8	coos: 7284	coos: 0.324	2550.9	7284	[27, 105]
Marsh wren	Unk.	syllables: 248	syllables: 0.09	23.8	248	[50, 107]
Mouse	4	syllables: 34124	syllables: 0.018	25277.0	133	[76]
Song sparrow	Unk.	syllables: 258	syllables: 0.105	32.8	258	[50, 107]
Swamp sparrow	616	elements: 97513	elements: 0.021	4571.1	1867	[21, 97]
White-rumped munia	44	syllables: 109851	syllables: 0.05	17118.5	169	[4]
Yellow warbler	Unk.	syllables: 246	syllables: 0.078	21.4	246	[50, 107]
Zebra finch	6	motifs: 18028<br>syllables: 65892	motifs: 0.443<br>syllables: 0.105	8799.9	18028	[99]
Zebra finch	46	elements: 3347	elements: 0.153	1365.0	3347	[24, 28]
