# Supplementary material for: Finding, visualizing, and quantifying latent structure across diverse animal vocal repertoires
Source: PLoS Comput Biol. 2020 Oct 15;16(10):e1008228. doi: 10.1371/journal.pcbi.1008228 (PMC7591061; doi:10.1371/journal.pcbi.1008228)
Supplement: S2 Table — For more information see Elie et al. [24, 28]. (PDF) [file pcbi.1008228.s008.pdf]

variable name	feature	feature type
fund	Mean F0 (Hz)	f0 features
cvfund	Coeff. var. F0 (0-1)	f0 features
maxfund	Min. F0 (Hz)	f0 features
minfund	Max. F0 (Hz)	f0 features
sal	Pitch saliency	f0 features
meanS	Spectral mean (Hz)	spectral features
stdS	Spectral std. (Hz)	spectral features
skewS	Spectral skewness	spectral features
kurtS	Spectral Kurtosis	spectral features
entS	Spectral entropy (0-1)	spectral features
q1	Spectral Q1 (Hz)	spectral features
q2	Spectral Q2 (Hz)	spectral features
q3	Spectral Q3 (Hz)	spectral features
meanT	Mean time (ms)	temporal features
stdT	Time Std. (ms)	temporal features
skewT	Time Skewness	temporal features
kurtT	Time Kurtosis	temporal features
entT	Time entropy (0-1)	temporal features
