# Supplementary material for: Finding, visualizing, and quantifying latent structure across diverse animal vocal repertoires
Source: PLoS Comput Biol. 2020 Oct 15;16(10):e1008228. doi: 10.1371/journal.pcbi.1008228 (PMC7591061; doi:10.1371/journal.pcbi.1008228)
Supplement: Attachment — Submitted filename: avgn_r1_reviewer-response.pdf [file pcbi.1008228.s011.pdf]

Dear Dr. Theunissen and Dr. Gershman,

Thank you very much for the opportunity to revise our manuscript, titled *Latent space visualization, characterization, and generation of diverse vocal communication signals* (#2019-19406). We greatly appreciate the constructive comments from each of the reviewers, and have revised the manuscript extensively in light of their suggestions. Major revisions, reflecting the concerns of multiple reviewers, are described below. Following this overview, we provide a point-by-point response to each of the reviewers' questions and concerns. We hope you agree that the end result is a much-improved paper that is now ready for publication.

We believe we have adequately addressed concerns brought up by all three reviewers.

Major changes

- We removed the Neural Networks / generative models section as was suggested by R3.
- We changed the title to "Finding, visualizing, and quantifying latent structure across diverse animal vocal repertoires".
- We added a section introducing graph-based dimensionality reduction.
- We added a section on choosing features to represent vocalizations, making a comparison with predefined acoustic features.
- We quantified comparisons in several sections (individual identity, species identity, sequential organization).
- We added a section discussing hierarchical density based clustering (2.3.3) as was suggested by R1, R2.
- We added a section comparing UMAP features to traditional features in swamp sparrows (2.3.4) as was suggested by R3.
- We added a section comparing hand labels to HDBSCAN labels on their ability to model sequential organization, quantifying comparisons using a Hidden Markov Model framework (2.3.5). As was suggested by R1 (point 1.13) and R3 (3.22).
- We added an explicit "Limitations" section to the discussion to discuss points made by all reviewers.
- To accommodate these changes, we reordered several sections of the manuscript.

Sincerely,
Tim Sainburg

Point by point responses

Editor remarks to Author:

First I am very sorry that the review process took this long! Your paper came during the winter break and once we found reviewers they also took a long time to finish their review. As you will see they agree that the paper and the UMAP approach you use is useful for analyzing sounds.

- All reviewers raised some questions that are mostly about clarifications of the methods and that I believe you will be able to address easily (more details and a more "even level" presentation of all the methods).
  We substantially modified the main text adding details based upon the specific points of the reviewers.

- There are also some questioning on the limitations of time-frequency representations (spectrograms) that are more open-ended questions but that do require attention.
  We address these questions in the text (Section 2.2), as well as a limitations section that we added to the discussion (Section 3.1).

- Maybe more significantly, your paper reads more like a very detailed tutorial for UMAP than a particular result.
  We agree that UMAP plays an important role in this paper. We rewrote sections of the paper to discuss why we currently use UMAP but emphasize that the primary interest in our current work are latent-variable models more generally. Additionally we spend more time unpacking UMAP, and discussing its relative merits as a current state-of-the-art model, we hope that our paper now reads as being a more general discussion of the utilities of latent models, pointing towards many of the potential uses of such models.

  I believe it will be very useful for anyone who will be analyzing/categorizing natural sounds.

Reviewer 1

This paper presents an overview of applications of deep latent representation learning to the study of vocal communication. The paper consists of three main parts. The first uses a recently introduced nonlinear dimensionality reduction tool (UMAP) to visualize structure in a large number of animal vocalization data sets. The second part reviews neural network methods that allow generation of samples from latent representations. The third part illustrates the application of one such method to behavioral and neurophysiological experiments in the starling.

This is an unusual paper, in that it is sort of mid-way between a review article and a research article. There are plenty of original results, but they are often impressionistic, given by visualizations that the reader is invited to inspect. In other cases (in the third part of the article), the results are somewhat anecdotal (and it is clear that a more thorough set of related results is being prepared for a dedicated research article). The paper feels more like an extended lecture than a typical modern-day scientific publication.

That said, I liked it. I found the approach refreshing and I liked the diversity, and found plenty of food for thought. The clusterability result of Figure 1 was cool, as was the comparative lack of clustering for speech (Figure 4). I suspect many people will enjoy reading this.

1.1. But there are some costs to the approach – for instance, the treatment of deep generative models is pretty brief, often at the expense of clarity. I think one could carve off part one of the paper and have enough for a substantial contribution. But I also think a paper with the current structure

is viable (though it will need to be longer in places in my opinion). Because this is not a standard scientific paper with a question, approach, results and conclusions, my comments are mainly geared towards improving the presentation (more along the lines of what I would give for a review article).

We substantially restructured the paper, increasing length in areas that Reviewer 1 suggested. Instead of carving off part one of the paper, we removed the neural networks section, and are expanding and preparing that section for a separate manuscript.

Major comments:

The paper is very well-written. It feels like a lot of care was taken in the writing, and it shows. The introduction is excellent – clear and accessible.

1.2. The paper leans heavily on UMAP, and could benefit from a deeper description of it, either at the end of 2.1 or the start of 3.1. As it stands I think someone who doesn't know about such methods will have a hard time contextualizing the results. I would like to see some equations and theoretical motivation.

We added a more substantial introduction to UMAP in which we provide theoretical motivation for UMAP in relation to other methods (Section 2.1) In addition we added a figure (Fig 2) showing how UMAP preserves structure in relationship to tSNE, MDS, and PCA.

1.3. In general, there is often a mismatch between the expertise needed to understand different parts of the paper. The paper seems aimed at the animal communication community, and so should have enough explanation throughout to be intelligible to someone from that community.

In the current revision we have tried to make parts of the paper that we found to be less accessible to members of the animal communication community without backgrounds in machine learning or signal processing. In particular we kept equations in the methods, and high level descriptions in the results.

1.4. Similarly, there is heavy reliance on HDBSCAN, but almost no information about it. Most readers will want/need more.

As with 2.2, we added a several paragraphs discussion HDBSCAN in relation to more traditional methods like k-means.

1.5. Section 3.3 is similarly too brief. There are many places where an additional phrase or sentence is needed to explain the underlying idea. Here are a few places that I noticed:

1.5.1. -line 49 – the regularization loss and its relation to the latent distribution

1.5.2. -line 439 – the reconstruction loss in latent space

1.5.3. -it would be helpful here to refer to the variables in the figure, which I don't believe are mentioned

1.5.4. -line 451 – adversarial loss function

1.5.5. -lines 451-452 – “train data…” – unclear – what problem does this solve?

1.5.6. -line 458 – costly in what sense?

We removed this section, but thank the reviewer for their comments, which will be taken into account for the manuscript focusing on this section.

1.6. There are a lot of figures, and they are frequently referred to out of order (and some are not referred to at all). Most journals require that figures, and individual figure panels, be referred to in order, and for good reason – it is much easier for the reader to be able to move through the text and figures in a coordinated way. This paper had pretty disjointed figure references.

We reordered figures as well as sections of the manuscript to discuss each figure in a more understandable order.

1.7. The article relies heavily on the spectrogram, but does not discuss the potential issues in mapping from spectrograms to audio. Griffin and Lim often produces unavoidable artifacts (because some spectrograms do not correspond to a physically realizable sound signal). I would expect this to be a limitation of what the authors are doing in the last section. We should be told what they sound like, and also whether the synthesized signals produce latent representations that are close to those intended (which might in principle not be the case – the spectrogram of the generated audio signal is probably not going to be identical to the sampled spectrogram). There are in principle ways that this could be handled better, e.g. by optimizing the waveform rather than the spectrogram to produce the desired latent representation. Worth some discussion.

We removed this section of the manuscript, but are including a section specifically about spectrogram inversion in the neural networks manuscript.

1.8. Related to point [2.12], the last section made me wonder whether the birds would produce similar behavior if trained only on the original exemplars. How much of the behavior is due to training on the synthetic morphs? If the synthetic sounds have artifacts, one might not expect great generalization from the original sounds to the interpolated sounds. But if similar results were obtained after training only on the originals, that would be impressive.

We have removed this section from the current manuscript.

This is an interesting question, and one that we are pursuing to some extent in an in preparation paper. Since all of the behavior in the paradigm outlined in 3.3.2 is ‘reinforced’ it would be difficult to get enough data without rewarding the birds using just probe trials. We have some preliminary results on neural populations responses of naiive birds however. These results show smooth neural responses across morphs. We have yet to rigorously compare the responses of trained and untrained birds, however.

Minor comments:

1.9. Line 104: “control control” – typo

We fixed this typo.

1.10. Line 169: “differentiable” seems like a poor choice in a paper that uses a lot of gradient descent. “discriminable” might be preferable.
We made the suggested substitution.

1.11. Figure 3: caption is in wrong order
We adjusted the caption.

1.12. Section 3.1.3 – why is HDBSCAN not run on raw spectrograms?
HDBSCAN is not run on the raw spectrogram because of issues with dimensionality. In the FAQ for the python HDBSCAN implementation (by the main author of UMAP) it is mentioned, for example, that 50-100 dimensions is the recommended upper bound to use with HDBSCAN. We added HDBSCAN on 100 PCA dimensions as an additional comparison (Section 2.2.3, Fig 10B).

1.13. Lines 262-264: can this effect be quantified in some way, rather than left for subjective evaluation?
We added a substantial section of the new revision of the manuscript dedicated to this, corresponding to Figure 14.

1.14. Line 292: “Projection section” – where is this?
This is a section in the methods, we added a hyperlink to all section references.

1.15. Figure 8 caption: The bits on panels M and N were unclear and hard to follow.
Now Fig 15. We added sub-labels to M and N for this figure.

1.16. Line 356 – missing parenthetical
We added the missing parenthesis.

1.17. Line 473 – please give the motivation for the J-diagrams
We removed this section, but added an explanation and motivation for the J-Diagram for the next manuscript.

1.18. Line 513 – “recording from extracellularly” – typo
We removed this sentence.

1.19. Figure 15E is not mentioned in the text that I could see. What should we conclude from the comparison of 15E and 15F? And why are we not shown the similarity in the latent space in addition to the spectrogram? I might expect the similarity of the spectrograms to not be particularly well behaved, so maybe 15E is in fact showing similarity in the latent space?
We removed this figure. It would be a good idea to show the divergence between similarity in latent space from similarity in spectrogram space. We will consider that for the next manuscript.

1.20. I was surprised not to see mention of texture synthesis approaches, which are the one prior case I know of where realistic-sounding natural sounds (including animal choruses) were synthesized from rich latent representations. Some of the same things proposed here were successfully pulled off in that domain (e.g. experiments with morphs – see McWalter and McDermott 2018). Seems worth mentioning somewhere.

We will add reference and discussion of McWalter and McDermott as well as other texture synthesis algorithms and approaches in the neural network manuscript.

1.21. I would think that an obstacle to scaling up the proposed approach is the need for relatively clean audio recordings. This seems worth commenting on.

We added two sections: (1) a Limitations section in the Discussion (Section 3.1), and (2) a section of the methods dedicated to noise reduction (Section 4.2). We also added a comparison between projections with different signal to noise ratios (Fig S6).

1.22. Figure 16 caption: "dynamically a noise threshold" – typo

We resolved this typo (now Fig 19).

1.23. Line 635 – why is there bandpass filtering? What are the parameters?

We updated the text to say that parameters are based upon normal frequency ranges of species vocalizations (lines 837-848). Bandpass filtering is generally used to segment spectrograms, and is not necessary when Mel scaling is performed within a frequency band.

1.24. Lines 637-638: confusing. What is a "Mel filter", and how does it produce a logarithmic scaling?

This line read, "*Either frequency is logarithmically scaled over a frequency range using a Mel filter, or a frequency range is subsetted from the linearly frequency scaled spectrogram*". We rewrote the line to explain Mel-scaling (lines #837-848).

1.25. Lines 644-651: unclear – equations would help. What does "sampled as a single point" mean?

"Sampled at a single point" was referring to the correspondence between a single window, and a single point in the projection space. Instead of adding equations, we added text and a figure to help understand how rolling window projections are done (lines #857-861; Fig. 20).

1.26. What was the "set length in milliseconds"?

This line read, "The rolling window is a set length in milliseconds and each window is sampled as a single point to be projected into latent space".

The set length in milliseconds is the window size (e.g. 1ms, 20ms, 100ms in Fig 15). We updated the text to make this more clear (lines #857-861).

Reviewer 2

Summary and major comments

2.1. In this paper Sainburg et al are reviewing and describing the applications and usefulness of state-of-the-art machine learning technics for representing and analyzing vocal communication signals with no a priori assumptions on the informative bearing acoustic features of vocalizations. They use a recently developed dimensionality reduction method (UMAP), finding latent variables in that space. They show how projections of sounds used for communication in this latent space uncover the organization of various large datasets of vocalizations recorded in dozens of different species.

The authors are showing two major applications of UMAP: 1. on discretized vocal elements or in other words, on audio recordings already segmented into individual vocal elements such as syllables or phonemes; 2. on continuous bouts/sequence of vocalizations such as a bird or a mouse song. Finally, in the last sections, the authors quickly present various generative models of vocalizations based on neural networks that can be used to generate artificial vocalizations that respect the statistics of the vocalization repertoire, such as morphs between two existing real vocalizations. They show the results of behavioral and neural experiments in Starlings that aim to test the perceptual boundaries of this species with such morphs.

Through graphical representations of the projections, the authors are showing how known properties of segmented vocal elements, such as the presence of identity coding acoustic features, are nicely displayed in the UMAP latent space. They also demonstrate how UMAP combined with a hierarchical density-based clustering algorithm (HDBSCAN, developed by McInnes et al) can achieve unsupervised categorization of song syllables as well as, if not better than a trained human observer, in the sense that new sub-clusters, revealed by this unsupervised method, seem to indeed correspond to distinct syllable types as revealed by their different transition probability in the bird songs. The application of UMAP on non-segmented data, with projections of vocalization bouts as trajectories in the latent space rather than individual points for each individual vocalization element, is an interesting approach that is resolving one major problem in the analysis of animal communication that is often addressed using some more or less reliable heuristic: segmenting sequences in meaningful elements for the considered species. The continuous projection method seems a promising avenue for species in which the segmentation cannot be easily inferred from the structure of silence durations, behavioral observations or play-back experiments.

The originality of this article lays in the usage and application of very recent mathematical methods to bioacoustics. Along with all the material provided under Github, I can see how this article could be very useful for researchers working in animal communication to access and use computational methods that they might not have even think of otherwise and give ideas of new analysis. Moreover, the authors made a particular effort to apply these methods to a multitude of datasets, illustrating their power to an impressive diversity of questions in animal communication. However, the present lay-out of the paper set it half between a research/method/review paper. If I had to choose, I think I would stress more the method aspect to it, increasing the didactic aspect by giving more methods details (explaining/describing briefly UMAP and HDBSCAN, giving more metrics for quantifying the results of the projections, giving equations for the quantification metrics already used (e.g. Comparing algorithmic and hand-transcriptions section)). I would also add a short table of content at the beginning of the paper such that the reader can see the organization of the article and easily navigate it. As such it reads a little too much as a catalog of interesting, but “glimpsy” results.

Broadly, we added large sections of text to give more in depth discussions of different methods, as well as to give equations and explanations for different quantification metrics where available.

We included a Table of Contents in the PDF file and would be happy to include it in the final manuscript the editor/journal would allow us to include it.

2.2. Finally, the last section of the article 3.3.2, illustrating how generative models can be used in animal behavioral and neurophysiological studies, presents data that are referenced as being presented at a conference but seem unpublished. As such, there should be a section in the method of the paper regarding animal training, handling, fasting, surgeries, procedures, protocol etc…

2.3. We removed the generative models section of the manuscript, and are preparing a new manuscript which expands upon this section. We will include those protocols in the new manuscript.

<u>Line by line comments</u>

2.4. **Line 80-85**: I agree that an alternative approach to cherry-picked engineered features designed by humans such as pitch, duration is necessary, and data driven features are the most attractive. PCA can indeed be a good dimensionality reduction as long as it is applied to a representation of the data that is not already doing a strong compression. In the case of ref 24 (acoustic analysis of Humpback whales), PCA was applied on engineered features, measurements made on spectrograms of vocalizations. Such an approach is not resolving the problem pointed by authors line 76-79. PCA applied to a conservative feature space, such as well-designed spectrograms, has been implemented in previous studies, these might be better references (Mouterde et al, 2014, Plos One; Elie & Theunissen, Animal Cognition, 2015). I generally see DFA as a supervised classifier and while I understand the statement of the authors regarding its ability to extract, in a data-driven manner, the features that distinguish a certain categorization of the data, it all depends on what input is used for the DFA. If the parameters used are once again human-picked measurements as was done in the analysis of the Marmoset repertoire, then the problem of human-oriented results stay the same. Here again, a non-apriori feature space on which to perform the DFA could be the vocalization spectrograms as projected in the first dimensions of a PCA (see Elie and Theunissen, Animal Cognition, 2015).

We added two sections. to the beginning of the manuscript discussing both latent projection techniques such as PCA, t-SNE, and UMAP (Section 2.1), as well as predefined acoustic features, as opposed to spectrograms (Section 2.2). In addition, we added a Limitations section (Section 3.1), in which we discuss the difficulty in determining the "distance" between two representations of vocalizations (lines #709-729).

We removed the statement about DFA, as we agree that the wording of the previous version of the manuscript gave the impression that we considered DFA an unsupervised technique.

2.5. **Line 103-104** What do you mean by "high degree of control in real-world experiments"? What are real-world experiments?

We removed this sentence, as it related to the generative sampling section removed from the current revision of the manuscript.

2.6. **Legend Figure1:** maybe you want to prefer the word "segmented" to "discretized"

To what type of category do the categories referred to for each dataset correspond? Acoustic

categories made by the authors? Meaning categories made by inference from behavioral observations? Identity of the vocalizer?

This is the figure of UMAP projections over different vocal repertoires (now Figure 4).

We updated the figure caption to use the word segmented, and to reflect that these are acoustic categories over individual vocalizations defined by the authors of the original dataset .

2.7. **Line 157-175:** Which metrics can be derived from the latent projections to quantify within and compare across species the potential for identity coding?

Here you selected vocalization types that we know by behavioral observation/playback experiments are used for individual recognition, would you have found that structure if you had been given the repertoire without any indication?

We updated this text to be explicit about prior results with the call types we selected for these analyses (lines #240-263). In addition, we quantified these distinctions using a clusterability measure (silhouette score) that takes into account ground truth categories (as opposed to the Hopkin's statistic which looks only at how clustered the data are, and not how well those clusters correspond to category labels). These methods are outlined in the methods (Section 4.6).

2.8. **Figure 5:** I'm curious about the individual contributions of UMAP and HDBSCAN to the clustering "performance" obtained with the combination of both methods. Did the author tested HDBSCAN/spectrogram? Can the contribution of each be quantified?

This figure is now figure 10.

We would have liked to test HDBSCAN directly on the spectrogram, but the dimensionality of the spectrogram is too high to perform HDBSCAN clustering. In lieu of this approach, we applied HDBSCAN to the maximum number of PCA dimensions that the author of HDBSCAN recommends HDBSCAN can work on (100). The results have been added to Table 1 and Figure 10.

2.9. **Figure 8:** First and second row of the figure are said to be the same but color coded by time (upper row) or hand-labels (lower row), however there seem to be missing points in the second row, why is that? Can a mention of this difference be explained in the legend of the figure?

This is the Bengalese latent trajectories figure (now Fig. 15). The missing points are points in the trajectory that are not part of a syllable (unlabelled). We added a mention in the figure caption.

2.10. Also, the legend of what I guess being the hand-labels colors in M and N is missing.

We added sub labels to M and N.

2.11. Finally, it could be useful to describe the colorspace projections of the UMAP projections in the methods, instead of having to dive into the code. Would help to understand what we're looking at in terms of color scheme.

We added a description of how colorspace projections were done in the Projections section of the methods (lines #517-519).

2.12. **Line 674-681:** Comparing algorithmic and hand-transcriptions section: The equations of the various metrics should be given.

We added the equations for the various metrics in the clustering section of the methods (Section 4.8).

2.13. **Section 3.1.4:** I understand the power of UMAP and HDBSCAN for automatically inferring the syllables categories from the data, but I don't see the extra degree of abstraction that one gets for the sequential organization of syllables with the projection in latent space.

This was the "abstracting sequential organization" section. It has been renamed to "Abstracting and visualizing sequential organization (3.3.5).

We agree that an extra degree of abstraction is not necessarily available from using our technique over any other hand labelling or algorithmic labelling scheme. The power of this method is having a visualization method to compare the abstracted model across element categories to transitions between individual elements. We added reference to this in lines 422-424.

2.14. **Figure 15 E and F** how is the similarity measure calculated?

This figure was removed from the manuscript (the answer is cosine similarity).

Minors comments:

2.15. **Line104 "control"** written twice

We fixed this typo.

2.16. **Line 124: "Supplementary"** should be supress

We fixed this typo.

2.17. **Line 197: To compare**

We fixed this typo.

2.18. **Figure3:** legend miss-ordered

We fixed this typo.

2.19. **Line 235:** sequences **of** a few

We fixed this typo.

2.20. **Line 253:** you probably want to refer to figure 7 D **K**?

We adjusted this paragraph to refer to each panel in Fig 7.

2.21. **Line 318:** a "strong hue"? a similar hue ?

We adjusted this sentence to read "similar hue".

2.22. **Line 338:** reference to figures inversed (Finch -> 8; starlings -> 9)

We fixed this typo.

2.23. **Line 343:** Wouldn't 8C instead of 8I be the good comparison here, in terms of color labelling?

The sentence read, "To visualize the vocalizations as a sequence of discrete elements, we plotted the entire sequence of USVs (Fig. 10I), with colored labels representing the USV's position in the reordered similarity matrix (in a similar manner as the discrete category labels in Fig. 6E)". We changed the reference to Fig. 12E, which shows discrete labels over time in a similar manner to Fig 17I (line #574)

2.24. **Legend figure 10**: the first and second **USV** dimensions are plotted as color dimensions -> the first and second UMAP dimensions are plotted as color dimensions?
We fixed this typo.

2.25. **Line 417**: remove "In the"
We removed this section.

2.26. **Line 448**: missing closing parenthesis
We removed this section.

2.27. **Line 448**: add the abbreviation here to define MDS-AE
We removed this section.

2.28. **Legend figure 12**: MD-AE - > MDS-AE; LSTM is not defined, Various D_x variables are not defined.
We removed this section.

2.29. **Legend figure 16**: a word is missing in "The algorithm dynamically a noise threshold based upon…. »
We updated this to read "the algorithm dynamically defines a noise threshold …".

Reviewer 3

Sainburg et al apply UMAP to bird song. This allows bird song syllables - time series of various lengths, often with considerable spectral complexity - to be projected into a small number of dimensions. They apply this idea to syllables and sequences of syllables. In addition, they use latent space models (this time with neural networks) to generate syllables from a data-set. In my opinion, this is the more novel and interesting part of the paper. Bird song is a major model for the neuroscience of learning, cultural evolution in non-human animals, and communication. Methods to improve our ability to computationally compare learned songs are therefore extremely valuable.

I feel that this paper represents an interesting advancement of this topic. I am not convinced by all the authors arguments about how their approach removes assumptions (see below), but nevertheless it is a valuable step in the right direction for the field.

General comments:

I was unfamiliar with UMAP before reviewing this manuscript, and have read through the documentation at this website: https://umap-learn.readthedocs.io/. This has led to some questions that I was unsure of from the manuscript:

3.1. (1) In the Methods, I found that there were not enough details in the Projections section. According to the documentation, there are quite a few settings to be made for UMAP (learning rate etc).
We added a discussion of the main parameters used in UMAP to the main text (lines X-Y). In all quantitative comparisons we used the default parameters. In some plots, we changed the "min_dist" parameter to aid in visualization of variability. We now mention that in the main text (lines #158-172). We also added a "Limitations" section to the discussion, where we also discuss parameterization (lines #740-745).

3.2. (2) How was the data entered into UMAP? I understand that the data for a particular syllable consisted of the power values from the cells of the 32 x 32 transformation of the spectrogram. Is it the case that each of these values consisted of a separate "dimension" of the data entered into UMAP?

That is correct, each time-frequency bin is treated as a unique dimension of the spectrogram. This is discussed in the methods (Section 4.4).

3.3. (3) If so, then would it be correct to argue that the comparison method underlying this paper is - ultimately - a form of spectrogram cross-correlation? The more similar the power values of cells in the two spectrograms, the closer they will be projected together into the low-dimensional space?

This leads me to my main critique of the manuscript, as I understand it. The original computational study comparing bird song was the spectrogram cross-correlation paper by Clark et al 1987. In that paper, spectrographic cross correlation was used to generate a dissimilarity matrix between the units of song compared. Then principle coordinates was used to project this into low-dimensional space. If I understand your work correctly, you have essentially updated this approach by using UMAP instead of principle coordinates. This provides some advantages (below), but the ultimate measure dissimilarity is rather similar to spectrogram cross-correlation. Is it not true that you could have used any other measure of song comparison (e.g. Sound Analysis Pro, DTW, parametric etc) as the input to UMAP (especially because according to the docs, UMAP will accept N x N dissimilarity matrices…)?

This is relevant because cross-correlational methods do make quite strong assumptions about what constitutes similarity between acoustic signals. And therefore, if I have understood your method correctly, I disagree with your characterisation of your method using unusually few assumptions of the data. For example, your methods make assumptions about the relative weighting of frequency versus temporal shifts in that once a change has been made large enough to shift energy into a different cell in either domain, then similarity will rapidly drop-off. For another, you chose to pad syllables at the end to make all spectrograms the same length - this would give different results from padding at the beginning. Cross-correlational methods also have other problems: they tend to be "brittle" in that as soon as there is no overlap between spectrograms, then similarity will drop to zero, and it can be rather hard to predict when this happens. From what I can tell, the same would be true in this case, unless UMAP is able to correct for it. So for example, imagine that two chevron-shaped notes were sampled, of identical 'shape' on the spectrogram, but separated by 500Hz in absolute frequency. I would imagine that no similarity would be found between them using this new approach… unless enough additional similar notes are also sampled which are intermediate in structure (in which case the nearest-neighbour approach used by UMAP may reconstruct the correct relationship between the two original notes.

We thank the reviewer for challenging our claims and have taken this point into careful consideration in our revised manuscript. Importantly, we note that our approach is not "*essentially updated [the cross-correlation] approach by using UMAP instead of principle coordinates"*. To address this confusion, we added section 2.1, describing how UMAP works in comparison to methods like cross-correlation, and section 2.2, in which we discuss how to represent distance as the input to UMAP. We also added a Limitations section to the manuscript where we discuss these issues.

To be clear, while UMAP and cross-correlation can both be used to quantify similarity, they are fundamentally different methods, based on mathematically distinct ideas. The UMAP algorithm is comprised of effectively two steps: (1) build a topological representation of the dataset (a finite set of points) (2) find a lower dimensional embedding of the dataset that preserves the invariant topological representation (i.e., the relationships between points) of the higher-dimensional dataset. The underlying assumption of UMAP is that the original data space (spectrogram space here) is a warped representation of the true manifold underlying the data (which is uniformly distributed). Therefore, distances (similarities) in the topological space are inherently different than distances in the original data space. Simple cross-correlation of spectrograms assess similarities in the raw data space, where small fluctuations may or may not be meaningful, and for this reason can be very “brittle”. In addition, unlike PCA which finds dimensions that maximize variance over the full data set, UMAP emphasizes variance both within *and* between clusters (i.e. global and local structure).

A good example to help consider this important distinction is ISOMAP. ISOMAP is one of the earliest topological manifold learning algorithms (Tennenbaum et al., 2000). The underlying algorithm is very similar to UMAP, t-SNE, and other graph based methods. ISOMAP computes a graphical representation of the dataset, and then finds an embedding in dimensional space that preserves the structure of the graph. In fact, the embedding that ISOMAP uses is effectively multidimensional scaling (MDS), the algorithm used in Clark et al., (1987). The difference between ISOMAP, and Clark et al.’s approach, is that in ISOMAP, the geodesic distances between points are the distances used to find a lower-dimensional embedding, rather than distances in Euclidean space. This distance, defined through topological structure, is qualitatively different from cross-correlation between spectrograms. We added an explicit comparison and discussion about the differences between principle coordinates, principal components, and graph-based dimensionality reduction in Section 2.1 (and Figure 2).

Still, to build this topological structure, one needs an initial representation of data, and a metric by which we can compute the distance in the original dataspace between vocalizations. We agree that spectrograms are one of many ways to represent the data, and are not, in and of themselves, “the data” (as the previous version erroneously stated). Thus, we added a discussion of the ways that we can compute the distance between vocalizations in the section 2.2 (lines #170-172), as well as a discussion of the more fundamental problem of considering the “distance” between two vocalizations in the Limitations section of the conclusion (lines #709-739).

3.4. (4) The UMAP documentation suggests that the algorithm is primarily to be used for visualisation, and even its authors say that its use for clustering is “somewhat controversial and should be attempted with care”. There is no discussion of this potential limitation in the manuscript. One issue is that UMAP may tend to generate artificial clusters.

We have had substantive discussions with the authors of UMAP about clustering and HDBSCAN (the lead author of UMAP also wrote the main implementation HDBSCAN). The emphasis being made in the above statement is that clustering should be taken with care, not that clustering should not be performed on UMAP (or TSNE, autoencoder, or other non-linear) embeddings.

The artificial clusters issue has to do with a trade off between capturing global and local structure. We added a discussion of the potential concerns with clustering on UMAP and other

topological embeddings such as spurious clusters that arise from a fundamental tradeoff between preserving local and global topological structure in the limitations section (lines #122-128).

3.5. (5) One issue with your method is that, like other "whole spectrogram" methods, it may be particularly susceptible to background noise or reverberation. Did you test this? In Figure 1, it seemed to play a role in some judgements.

We added some discussion of noise throughout the manuscript (lines #686-700) and methods (Section 4.2). We also added a comparison between projections with different signal to noise ratios (Fig S6).

3.6. (6) In Figure 1, my impression was that the analysis did not perform well in cases where there were a large number of categories, but with some categorical structure. For example, in the case of swamp sparrows, the note examples in the top left has clear resemblance to the note examples in the bottom-right and in the bottom-left (in all cases, relatively long notes that descend in frequency at an increasing rate), despite these being in very different positions on the projection. There was a similar pattern with the Cassin's vireo. The two note examples in the top left show a small chevron followed by a more prominent upsweep in frequency. That same pattern is found in another note towards the bottom right - placed in a cluster that is very far away in the projection. In the case of starlings, there are several cases where highly harmonic chevron shapes predominate, yet these are projected to very different places. These results seemed to go very clearly against what I would predict from other analyses of similarity, or from subjective human judgment. What validation of these results can you provide? Is it possible that this method will effectively separate different note type categories, but still fail to reflect more global patterns of structure?

It is true that UMAP (or other topological embeddings such as t-SNE or LargeViz) forces a tradeoff between representing local and global structure, so that similar clusters may not be near to each other in embedding space (although UMAP performs better than t-SNE (Becht et al. 2018)). It is important to remember that these are projections, and distance does not correspond directly to perceptual similarity.

3.7. (7) Overall, I think the paper would benefit from clearer, quantitative efforts to compare the output of this analysis method with previous methods.

We added a number of quantitative comparisons and analyses over our current methods. For example, we added comparisons in (2.1) PCA, MDS, t-SNE, and UMAP representations of projections, (2.2) Predefined acoustic features and spectrograms (2.3) we quantified our comparisons across species for individual identity on the basis of silhouette score (3.4) we quantified our comparisons across species (e.g. power spectrum vs UMAP features) on the basis of silhouette score. We also compared clustering algorithms (KMEANs/UMAP over different feature spaces) on the basis of V-Measure. We quantitatively compared clusterability in projections on the basis of Hopkin's statistic. We quantitatively compared hand labels, hidden Markov models, and UMAP/HDBSCAN clustering on their ability to capture sequential organization on the basis of AIC.

If there is a specific method with which the reviewer would like to see a comparison, we would be happy to make a comparison.

3.8. (8) Section 3.3 seems to really be a different paper. The links between 3.1/2 and 3.3 are rather tenuous - the methods involved are really quite different. While I think both are interesting, I wonder whether a more focused and useful result could be generated by splitting this into two papers.

We ultimately decided to take the reviewer's suggestion, and are splitting off 3.3 into a second paper.

Specific Comments

3.9. Line 66-79 - while I agree with the thrust of this paragraph, is it not also the case that spectrograms are a particular and not particularly perceptually valid representation of the data (they are not "the data" as you say in the next paragraph). And its not immediately obvious that all features would be accessible to your method. For example, relative pitch or Wiener entropy are quite 'perceptible' to us ('melody' and 'spectral noisiness') but I do not see how your algorithm could detect changes in either.

As noted in 3.3, we agree with the reviewer. We added a section dedicated to representing vocalizations (section 2.2) and the difficulties in representing distance between vocalizations in the discussion (lines #709-739).

3.10. Lines 80-81 - there's an assumption here that the structure of the data (produced vocalisations) will reflect animals' perception. That's not necessarily the case - there might also be, for example, physical limitations on sound production which mean that the structure of the data deviates from how animals perceive it. In the case of highly clustered data (like learned bird song), there's an additional question of how much data you would need to collect before it provided the information needed to shape the manifold (for example: does the vocal repertoire of one zebra finch provide enough information to learn the structure of zebra finch vocalisations?). I don't disagree with your argument here, but believe that its own assumptions and limitations need to be fleshed out a little more fully.

This line reads, "An attractive alternative approach is to project animal vocalizations into low-dimensional feature spaces that are determined directly from the structure of the data."

As noted above, we agree with the reviewer. We left this sentence as-is, because, as the reviewer notes, they agree with the statement, it just needs to be fleshed out. We added some discussion of this issue throughout, specifically pointing to animals perception in places (e.g. lines #664-668)

3.11. Lines 85-86 - this seems like unsupported assertion. (Or maybe I don't understand better than what?)

This line was referring to the ability to represent data using non-linear transformations (e.g. extracting topological structure) as opposed to linear methods. We several paragraphs explaining this distinction (lines #113-147).

3.12. Lines 95-96 - I disagree with this assertion. I think that your method makes as many assumptions as any other method.

We revised this sentence to read "In this paper, we describe a class of nonlinear latent models that learn complex feature-spaces of vocalizations, requiring few *a priori* assumptions about the features that best describe a species' vocalizations". In the paragraphs above the referenced line, we discuss the difference between feature engineering and data-driven approaches. As we've clarified in the revised introduction, and sections 2.1 and 2.2 it is not our intent to propose a single method that is best for all analyses of all vocal repertoires. Rather, we are simply making

the point, as others have done (e.g. see (Elie and Theunissen 2016), paragraph 2), that the data-driven method requires fewer *a priori* assumptions than feature engineering approaches, for the simple reason that any feature targeted in the latter is an a priori assumption. This is not to say that *a priori* assumptions are bad: swamp sparrow songs may very well be fully explained by a few simple features, but using such features as a basis for classification is a very strong assumption. Without *a priori* knowledge of these features, one must either study the animal's communicative repertoire to develop expertise, or use a data-driven approach (i.e. the methods presented here or PAFs, section 1.2). The tradeoffs between feature-based and data-driven approaches in discussed revised section 2.2.

3.13. 3.1.1 - It is not surprising that species differ in their clustering tendency when the data-sets are so different: some are from just one individual while others are from very many. I didn't really understand the comment in the legend of Fig.1 about how the choice was made to use one or many individuals. In the case of swamp sparrows, individuals certainly have distinctive song repertoires (as much as zebra finches, for example). I think, for example, that had you sampled syllables from a population of zebra finches, you would have found little clear clustering.
I think this point applies to the comparisons made between birds and mammals and between birds and human speech (3.1.3) too.
We added a comparison across only datasets where individual identity was available (all datasets in Fig 8 except giant otter and gibbon; lines #330-339). We agree that there is nuance to this result that could only be rigorously addressed in a controlled study where large datasets exist of individual vocalizers and segmentations are performed on some atomic units of vocalizations that can be reasonably assumed to be similar levels of abstraction across individuals. Such a dataset does not exist. We still believe insights can be gleaned from our comparison. In this revision we emphasise the issues with making interpretations based upon this analysis in several points throughout the text (e.g. lines #342-343).

3.14. Lines 154-156 - these claims might also apply to naive cross-correlation methods too.
This sentence read, "These latent projections capture a range of complex features, including individual identity (Fig 2), species identity (Fig 3A,B), linguistic features (Figs 4, 18), syllabic categories (Figs 6, 5, 1, 7), and geographical distribution (Fig 3C). We discuss each of these complex features in more detail below."
We agree that vocalizations with similar features are often correlated. It is not our intention to say here that these methods alone capture structure in vocal data. In section 2.1, we added a discussion of the differences between the structure found by these methods.

3.15. Line 189 - In this case, the classification rate of the two whale species does not seem particularly high, and you refer to other work used to identify species. Can you compare your classification success with other methods?
In our initial submission, we did not compute the classification rate of the two whale species, we simply showed that the relationship appears to cluster in the latent representation. UMAP is not a classifier.
In comparing different algorithms for clustering, we want to be cautious not to create a set of "straw man" algorithms that our algorithm can "beat". As we are sure the reviewer is aware, unlike many areas of machine learning where clearly defined datasets and leaderboards exist to determine "state-of-the-art" algorithms and code are published alongside manuscripts, bioacoustics datasets often go unshared and code unpublished (for various reasons). For

example, in the only unsupervised click clustering paper we were able to find (published in PLOS Comp Bio; Frasier et al., (2017)) we were not able to find a code availability statement in the document or any implementation online, which was not unusual for bioacoustics until very recently.
We did add a quantification of the silhouette score, and compared it to the silhouette score of the spectrum alone, which is typically used for classification.

3.16. Line 195 - I don't think "dialects" is the correct term here - "regiolects" might be more accurate. In addition, the categorical perception in swamp sparrows is found within populations, not between populations.
This sentence read, "Some vocal learning species produce different vocal repertoires (dialects) across populations". We swapped the two terms throughout the manuscript.

3.17. Line 197 - typo compared -> compare
We fixed the typo.

3.18. Fig.3C and commentary - Unfortunately, the projection of swamp sparrow notes really doesn't match with any structure found in previous studies, so this result is hard to evaluate (also - you can get a clearer representation with a similar conclusion simply by plotting peak frequency change versus note length for these very simple notes). The good thing about swamp sparrow notes is that they are simple enough to capture even with just a couple of features, which might allow you to test the performance of your method. One thing I would like to know is whether the buzzy, frequency modulated notes that swamp sparrows sometimes use show up as a separate cluster or not.
We added an analysis of the swamp sparrow song we had available, comparing peak frequency change versus note length (Fig 11). We observe a fairly high degree of similarity between the two projections (also compare to Fig. S2 in (Lachlan and Nowicki 2015)). We did not have cluster labels for that dataset to compare HDBSCAN and clustering in the 4 parameters they use directly, but we do find that the clusters in our analysis match up fairly well. We encourage the reviewer to dive further into this particular example to look at specific features themselves.

3.19. Figure 3 - A and B are labeled the wrong way around in the legend.
We fixed this error.

3.20. Table 1: Finch should be labeled zebra finch.
This analysis used Bengalese finches, not zebra finches.

3.21. Appendix: in the case of swamp sparrow notes, there seem to be broad-band artefacts at the end of most notes.
We added some tapering to swamp sparrow notes to remove artifacts.

3.22. 3.1.4/3.2 Lachlan et al. 2013 (Current Biology) also used a method to abstract sequential structure from the structure of the data - looking for categories that maximised redundancy. It would be interesting to apply this method to your UMAP clustered data. This method was developed for data that was not clearly clustered…
Clustering using sequential information is a method we find very interesting. In the current revision, we used HMMs to cluster based upon sequential organization (Section 2.3.5 lines

#465-488). A similar method could be employed with less structured data, where emissions would be continuous rather than discrete.

3.23. 392: again, I really don't think you can argue that your method is less biased than other studies.
It is not our intent to be dogmatic regarding the benefits of data-driven vs. feature-based approaches. Each have benefits and we've tried to balance our discussion of those benefits in the context of presenting our methods (see revised introduction and section 2.2). This sentence reads, "These methods enable new forms of visualization, help improve understanding of the structure of animal communication, and yield an unbiased (or at least less biased) feature set from which analyses of vocal communication can be performed."
We provided our argument for why data driven methods are, in general, less biased than feature engineering in the introduction of the paper (lines #92-106) as mentioned in point 3.12.

3.24. Categorical perception experiment - categorical perception relies on stimuli evenly drawn from a continuous physical dimension being warped in perception. I am not completely sure you can claim that your latent representations are evenly reflecting a continuous dimension. The very fact that these methods allow non-linear dimension reduction is surely an issue here?
We removed this section.

3.25. 3.3.1 - I'm not entirely sure what the conclusion to draw from this analysis should be.
We removed this section.

3.26. Fig. 14 - the figure legend doesn't explain what d is… and I couldn't make sense of the labels above the figures, in light of the figure legend below.
We removed this section.

3.27. 3.3.2 - Playing devil's advocate a bit, but: in 14A and 15B, as you go from one syllable to another, it almost looks like a case of masking. In the top-left of 14A, for example, the "volume" of the top-left original is at maximum, and the "volume" of the top-right original is at 0. As you move along the top row towards the top-right, the "volume" of the top-right original is gradually turned up, and that of the top-left original is turned down. In the middle, both original stimuli are played at equal volume. If that is all that is happening, perhaps the subsequent behavioural/neurobiological results are fairly understandable?
We removed this section.

Methods:

3.28. I would appreciate a fuller and clearer explanation of how choices were made to log transform frequencies or not; and how spectrogram data were entered into the UMAP algorithm. It would be good to know how sensitive the results were to the choice of 32 x 32 bins for the spectrogram. How would 16 x16 or 64 x 64 differ?
We did not perform rigorous parameter sweeps across each of the datasets. We now mention this in lines X-Y. Certainly hyperparameters like this could be improved and modified to fit different species. We only used log scaling of frequency for the UMAP analyses. In the previous version we sometimes used linear scaling to improve reconstructions for spectrogram inversion. Linear scaling is not used in this version of the manuscript.

3.29. The details for the neural networks seem quite sparse. This reinforces my feeling that 3.3 belongs as a separate paper.

We removed this section.

Bibliography

Becht, E., McInnes, L., Healy, J., et al. 2018. Dimensionality reduction for visualizing single-cell data using UMAP. *Nature Biotechnology*.

Elie, J.E. and Theunissen, F.E. 2016. The vocal repertoire of the domesticated zebra finch: a data-driven approach to decipher the information-bearing acoustic features of communication signals. *Animal Cognition* 19(2), pp. 285–315.

Lachlan, R.F. and Nowicki, S. 2015. Context-dependent categorical perception in a songbird. *Proceedings of the National Academy of Sciences of the United States of America* 112(6), pp. 1892–1897.
