# Supplementary material for: Finding, visualizing, and quantifying latent structure across diverse animal vocal repertoires
Source: PLoS Comput Biol. 2020 Oct 15;16(10):e1008228. doi: 10.1371/journal.pcbi.1008228 (PMC7591061; doi:10.1371/journal.pcbi.1008228)
Supplement: Attachment — Submitted filename: PCB-AVGN-reviewer-response-round2.pdf [file pcbi.1008228.s012.pdf]

Dear Dr. Theunissen and Dr. Gershman,

Thank you very much for the opportunity to revise our manuscript, titled *Latent space visualization, characterization, and generation of diverse vocal communication signals* (#2019-19406). We greatly appreciate the comments made by all three reviewers and have revised the manuscript to address their suggestions.

Sincerely,
Tim Sainburg

Point-by-point responses:

Reviewer #1:

The authors have done a very good job in the revision of this manuscript that is now more focused around a same objective/story: the potential of latent projections for the study of animal communication. This paper is didactic and I like in particular the effort made with the new figures and sections of the paper (introduction on UMAP, discussion/limitations of the approach).
This will definitely be a very useful contribution to the field!

I spotted some typos that the authors will want to correct:

1.1. Line 68: suppress comma : Kershenbaum et. al., [1] -> Kershenbaum et. al. [1]
We fixed this.

1.2. Figure 1: in the legend correct: UMA,P -> UMAP
We fixed this.

1.3. Figure 3: revise legend: “(E) UMAP of spectrograms where the color for each syllables where color is the syllable’s average fundamental frequency (F) The same as (E) where pitch saliency of each syllable, which corresponds to the relative size of the first auto-correlation peak represents color.”
We fixed this.

1.4. Line 305: Fig5C -> reference to wrong figure
We fixed this.

1.5. Line 335-343: Text paced twice
We fixed this.

1.6. Line 360-380: Check consistency for spelling “v-measure” or “V-Measure”

We updated this to “V-measure”, as is used in the original publication.

1.7. Line 455: Fig13 D,J -> Fig 13C,J?
We fixed this.

1.8. Figure 14 : Error in legend reference to subplot C and D
We fixed this.

1.9. Figure 15: “The same plots as in (A),” -> “the same data as in A-C”?
We fixed this.

1.10. Figure 15: “The same plot as (D-E)” -> “the same plotS as (D-F)”
We fixed this.

1.11. Figure 15: Can you add the time color scale used in A-C at the bottom of spectrograms in M and N so we know what color represent the beginning/end of the bout?
We added text to the figure label (Figure 15) to describe the colors at the beginning/end of the bout.

1.12. Figure 20: Make sure you define “STFT” (not everyone might guess this is Shot Term Fourrier Transform)
We defined STFT (Figure 20).

1.13. Figure 16: “corresponds is” -> “corresponds to”
We fixed this.

1.14. Discussion about how you choose 200ms as the optimal size of the rolling window for Starlings?
We added a few sentences explaining the choice of 200ms time window (line #528).

1.15. Line 557: to categories -> “to categorize”
We fixed this.

1.16. Line 564-565: Specify what the distance metric is: Euclidean distance between latent projection vectors?

We updated the text to say "measured by the Euclidean distance between latent projection vectors" (line #516).

1.17. Figure 17E: Specify the size of the scale bar: 100ms?

We added the time length of the scale bar (Figure 17).

1.18. Line 596: "more smooth" -> "smoother"

We changed this.

1.19. Line 737: missing word: In this ?

We changed this to "Here"

1.20. Line 938: You probably want to remove that sentence: "The behavioral and neural data are part of a larger project and will be released alongside that manuscript."

We removed this sentence.

Reviewer #2 (we erroneously listed this reviewer as #3 in our previous response letter)

As with the first version of this manuscript, my overall impression of this paper is very positive: I think it pushes the field of bioacoustic analysis forward by engaging with new methods in machine learning.

The revisions to the manuscript have improved its clarity and focus. I still have some concerns, however, about details of the method and its performance in measuring differences between song units.

2.1. In the Introduction (or I think the rest of the paper), spectrogram cross-correlation is not mentioned at all. While I agree with the authors that there are significant differences between this older method and their approach, it also appears to me that there are clear and deep similarities. Both rely on a pixel by pixel comparison of the spectrogram without any extraction of acoustic features or contours. Cross-correlation has been an extremely influential method in the field, and its strengths and weaknesses have been widely debated. I think that it can only help the paper to discuss the similarities and differences between their method and cross-correlation, and I would strongly urge the authors to do so.

The methods we presented here are not mutually exclusive of other specific distance metrics, including cross correlation. In the previous revision of the manuscript, we added

a discussion of the different metrics of distance could be used to build a UMAP graph (lines #742-752):

> *"In principle, any distance metric could be used in place of Euclidean distance to build the graph in UMAP. For example, the distance between two spectrograms can be computed using Dynamic Time Warping (DTW) [87, 88] or Dynamic Frequency Warping (DFW) [89] to overcome shifts in frequency or time. Determining what notion of distance is most reasonable to compare two vocalizations requires consideration. When acoustic features are known to capture the structure of an animal's communication, either by careful study or explicitly probing an animal's perceptual representations of their vocal repertoire, the distance can be computed on the basis of those acoustic features. Here, we use Euclidean distance between spectrograms to build UMAP graphs, which we find is effective to capture structure in many vocal signals."*

The previous revision also included a section in which we used extracted acoustic features rather than spectrograms to compute distance in UMAP (Section 2.2, where we use PAFs from BioSound), and a section where we compared UMAP projections of distances computed over acoustic features extracted from Luscinia with swamp sparrow vocalizations in Fig 11D. In the code, we also provide an example of a Dynamic Time Warping distance metric.

To make it more clear that UMAP is not exclusively tied to the Euclidean distance between spectrograms as a metric for similarity, we added cross-correlation to the list of distance metrics mentioned in the discussion (line #747).

2.2. One aspect of cross-correlation that was very important to its success was the concept of a "sliding window": one spectrogram was moved across the other in the time domain until the correlation between the two of them reached a maximum.

I am concerned that no such alignment process occurs in this new method, and I am not sure that the graph-based approach of UMAP can always rescue it. Sorry that the following is rather crude, but I think it explains my concern most simply. Imagine two syllables with partial similarity - they both include a downward slide, but one begins with a short period of unmodulated high frequency (I will try to upload a sketch). Because of this, in your method, there would be very little overlap between the two spectrograms, in isolation, they would be judged as very different by UMAP. However, if there is a very good sampling of notes from the population, then we might expect plenty of intermediates between the two to exist, and it would be these intermediates that would allow UMAP to discover the similarity between the original non-overlapping two calls in geodesic distances. The question then becomes whether or not the sampled notes adequately cover the latent space so that such intermediates allow similarity between non-overlapping spectrograms to be discovered. One would therefore expect this method to perform well for non-clustered data, such as shown for many of the mammal examples.

In fact, it is my subjective feeling that this is the case, for example for the marmoset phee calls. But for highly clustered data, I think it would be very difficult for the method to discern global structure at all. And again, in the examples of more clustered data-sets, there seem to be more cases of unusual placement of syllables.

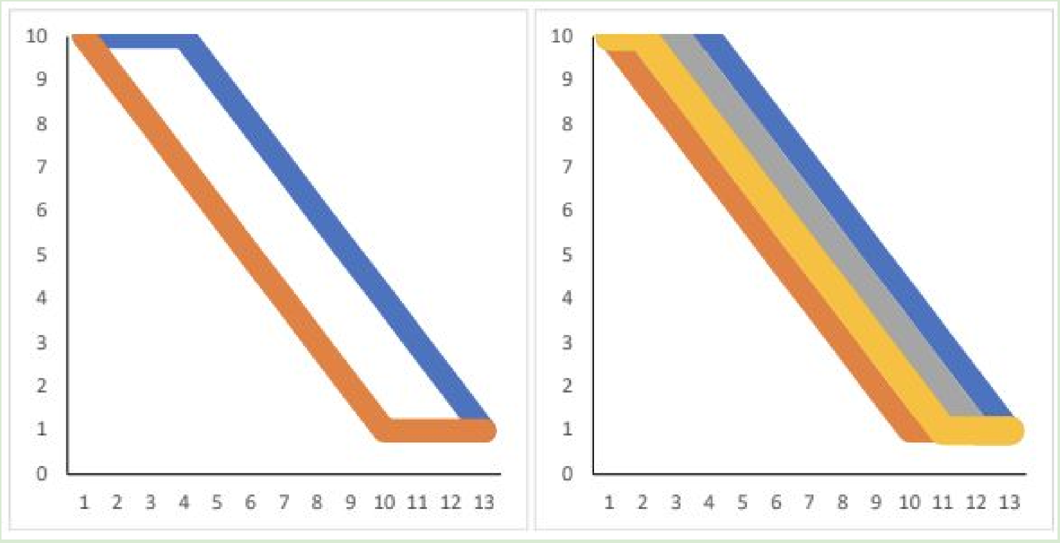

Pseudo-spectrograms. If two notes are sampled (left), the lack of overlap between them on the spectrogram will mean that their similarity is missed by the algorithm. If enough intermediates are sampled (right), then similarity between partially similar notes can be recovered from graph distances.

I do not know if there is any way to provide a statistic that will inform the user about whether or not the data coverage is adequate or not to prevent such problems. You do mention the issue of sample size in the limitations section, but it would be good if you could provide more details about how a user could judge whether the sample size is adequate or not.

I have thought about whether there is an easy solution to this, but only have a very crude suggestion. Would it be possible to enter each syllable into the analysis 10 times, with a different time lag each time? So the data entered to UMAP would be in a 42 x 32 pixels. The first time the spectrogram was entered, it would be aligned at the left of that grid (with the right-hand ten columns set to 0), and the last time, to the right of the grid (with the left- hand ten columns set to 0). Then to find a placement of the syllable, you would take an average of the position of those 10 entries.

To reiterate our remarks in 2.1, cross-correlation, as well as DTW, DFW, distance between PAFs, are all viable distance metrics that can be used with UMAP.

The section "Representing data and distance across vocalizations" (lines #722-752), directly addresses the issue raised by the reviewer. In it, we note:

> "*In principle, any distance metric could be used in place of Euclidean distance to build the graph in UMAP. For example, the distance between two spectrograms*

can be computed using Dynamic Time Warping (DTW) or Dynamic Frequency Warping (DFW) to overcome shifts in frequency or time."

The solution to the problem the reviewer proposes ("would it be possible…") would also be a reasonable metric to use, but rather than using time-shifted syllables as input to UMAP, a peak in cross-correlation could be used as the distance metric directly. We now note this specifically in the revision (lines #745-747).

"*For example, the distance between two spectrograms can be computed using Dynamic Time Warping (DTW), Dynamic Frequency Warping (DFW), or peaks in cross-correlations to add invariance to shifts in time and frequency between vocal elements.*"

2.3. In your reply to my comments, you state that UMAP is a projection, and that "distance will not correspond to perceptual similarity", especially when considering global similarity. While I understand that this method might not correspond precisely to the gold standard of perceptual similarity, I don't really understand this reply. Do you really mean that the method does not make any reliable measures of non-local similarity? If so, then surely it is only useful for clustering analyses, and most of the figures provided in the manuscript are misleading. If you mean instead that global measures of dissimilarity only correspond approximately to true dissimilarity, then I don't think you have addressed my previous comment. I think it will be very important to users to know how to use the types of projection that are generated by this method. Does it only allow reliable clustering of notes into types? Or can it be used to make broader scale quantitative comparisons between notes or syllables? I think this is a misunderstanding on my behalf, not a major point.

We should have been more specific in our reply, and noted instead that distance in the UMAP projection is not "guaranteed to correspond directly to perceptual similarity". This is of course true for all measures based on the physical structure of a stimulus, because perceptual similarity is a combined function of physical structure and behavior.
Our caution regarding the interpretation of distance for a UMAP projection does not mean that the method yields no reliable measure of non-local similarity. Indeed, one of the highlights of UMAP is its capacity to capture more global structure than, for example, t-SNE and LargeVis (McInnes et al., 2018), while avoiding the well-known pitfalls of methods such as PCA or MDS that only rely on global variance. We agree that the tradeoff between local and global structure is an important consideration. To make the distinction between the type of structure captured by UMAP more clear, we added a paragraph to the discussion (lines #722-766).

2.4. The main methods that are used in this field at the moment are, I think: (a) parameter-based comparisons, which this paper does indeed compare with for swamp sparrow song; (b) a form of linear time-warping used by Sound Analysis Pro; (c)

spectrogram cross-correlation; (d) a very distant fourth, various flavours of dynamic time warping. I think it would be useful to compare the analysis of zebra finch song with comparisons made by SAP - and in particular to see which provides better comparisons with human judgements of song similarity. This is because SAP is such a standard method for the zebra finch community, and if you'd like that community to use your method, such a comparison would be important. I also think it would be interesting to compare this method with Spectrogram Cross-Correlation, because, as above, I see this method as potentially a big improvement on that method.

As is mentioned in 2.1 and 2.2, while we primarily opted to use Euclidean distance between spectrograms as a distance metric, the methods we present here are largely orthogonal to distance metrics such as spectrogram cross correlation, DTW, linear time warping, and PAF-based distances. In the previous revision, we added a comparison with different underlying distance metrics for UMAP however (BioSound features versus Euclidean distances over spectrograms). To make this distinction more clear, in addition to the sections we added on different features as input to UMAP (Section 2.2), in this revision, we added to our discussion of distance metrics (lines #722-752).

2.5. One point I think you don't make (or at least don't make strongly enough) is that this method can be applied easily to large data-sets. To reliably extract acoustic features from data is either normally very time-consuming, or, if automated, not very reliable. Spectrogram-based methods are much superior in this regard, but the main method used for that, spectrogram cross-correlation, has problems in terms of the reliability of its comparisons. While I am not entirely convinced that all of these have been solved by this method (see above), it is clearly a big step forward. For applications such as the analysis of sequences, large data sizes are critical, and reasonably reliable, fully automated methods are very useful for the field.

The reviewer asks for more emphasis on the proposed method's ability to reduce the amount of time needed to extract features from data. We added mention of the utility of the methods discussed in this paper on large datasets on lines #136-137.

2.6. In a few places in the manuscript, there are claims that I think go a bit too far about biases and assumptions. In the Abstract (L10) and summary (L21), you argue that the method allows "comparative analyses of unbiased acoustic features". I'm not sure that's the best description of the method (since the point is not to extract features at all), but I definitely disagree with the use of "unbiased" here. This method definitely does have some a priori assumptions that impose biases on the result (even if we disagree about whether they are fewer or greater in number than other methods!). I appreciate the line that the authors' took in their response to the previous round of comments, but it would be great if they could check through the ms to make sure that it is always in-line with that.

We appreciate how the reviewer has read our use of the word 'bias'. To be clear, we are not using it synonymously with 'assumption'. Instead, by 'unbiased feature' we only mean a feature that the user does not define prior to the start of the analysis. It is not our

intent to imply that our method is free of underlying assumptions, or that ‘unbiased features’ are always preferable to ‘biased’ features (i.e., ones selected a priori). As the reviewer notes, we tried to integrate these points into the revised ms. To guard further against any mis-interpretation, we have modified the wording in the abstract and summary as suggested.

Detailed comments:

2.7. Line 10: “unbiased acoustic features” - I’m not sure that’s correct. (a) I’m not sure you have removed all “biases”, and (b) I’m not sure that’s even a well-defined goal.

See response to 2.6

2.8. Line 21: Similarly - not sure how you have demonstrated unbiased comparisons.

See response to 2.6

2.9. Line 78: But this method is equally shaped by unvalidated assumptions that create biases. You made a choice whether to log-transform frequencies, for example.

The line (now line #77) reads, “*Even in well-documented model species, characterizations of vocalizations are often influenced by human perceptual and cognitive biases*”. We contend that this statement is true and elect to leave the sentence unaltered. As noted in response 2.6, it is not our intent to imply that our methods are free of all assumptions. Rather, we only argue that our methods can be used to examine and compare vocalizations in instances where one either does not have access to or does not wish to use pre-defined acoustic features.

2.10. Line 330.. could you not include whether or not recordings were made from one single versus many individuals in this model? I feel that this comparison is fairly meaningless without that.

As is stated in line #331 (now line #344-346), the analysis is made over single individuals.

2.11. Line 849: Log-rescaled in time. Surely this means that variation at the onset of syllables is assumed to be more salient than variation at the end of syllables? What is the basis of this assumption?

Log-rescaled in time means here that the spectrogram is downsampled relative to the log of the duration of the syllable. We added the following text for clarification: “*(i.e. resampled in time relative to the log-duration of the syllable)*” (line #879). Log rescaling is performed so that shorter syllables are not under-emphasized due to low time-resolution. Thus, onsets are <u>not</u> more heavily weighted than other timepoints.

Reviewer #3: (we erroneously listed this reviewer as reviewer #2 in the previous response letter)

The revision of this paper is improved. I recommend publication after the authors address my comments as they see fit (all minor). I don't need to see the paper again.

3.1. The most substantial revision the authors should consider is to lengthen and improve the description of U-MAP at the start. I fear the description they give is still not sufficient to give someone who is not already familiar with such methods a sense for how/why it works. It would help to spell out what it means to find an embedding that preserves the structure of the graph.

We added a paragraph to 2.1 to go further into detail into how UMAP works, how it relates to t-SNE and what it means to find an embedding that preserves the structure of a graph (lines #161-180).

3.2. Another important point is that the authors should emphasize at the outset that U-MAP does not use labels. This is not made explicit on page 6. It is stated later on, but should be prominently mentioned earlier.

We now mention in the first paragraph of the dimensionality reduction section on page 6 that UMAP "is unsupervised, meaning it does not require labeled data" (lines #513-154).

I love the analysis of co-articulation. Very cool.

Other small things I spotted:

3.3. 163: "problematically" – wrong word

We updated this to "probabilistically"

3.4. 228: order of figure numbers is wrong

We updated the ordering.

3.5. 282-284: specify which version is shown in the figure (UMAP on spectrograms or power spectra)

We added text indicating the spectrogram (line #298)

3.6. 376-377: what is the difference between “sub-clusters of the hand labels” and “hand labels that are split into multiple categories”? unclear to me.
We changed the text to say “A high homogeneity and low completeness score indicates that algorithmic clusters tend to fall into the same hand-labeled category, but multiple sub-clusters are found within each hand labeled category” (lines #383-386).

3.7. 404: “similar level of overlap with position of notes” – confusing
We changed this to “similarly related to the position of notes within a syllable (e.g. first, second, third).” (lines #412-413)

3.8. 423: “element” – typo
We fixed this.

3.9. 557: “categories” – typo
We fixed this.

3.10. Fig 17F is blank
We updated high-resolution vector images corresponding to each figure alongside the manuscript.

3.11. 650: “behavioral: - typo
We fixed this.

3.12. 734-735: “Determining…” – something got garbled
Text

3.13. 737: “In this ,” – missing word
We fixed this.

3.14. 788: “deficit” – wrong word
we changed this to “issue with” (line #817).
