# Supplementary material for: Finding, visualizing, and quantifying latent structure across diverse animal vocal repertoires
Source: PLoS Comput Biol. 2020 Oct 15;16(10):e1008228. doi: 10.1371/journal.pcbi.1008228 (PMC7591061; doi:10.1371/journal.pcbi.1008228)
Supplement: Attachment — Submitted filename: PCOMPBIOL-D-19-02154_reviewer.docx [file pcbi.1008228.s009.docx]

**Summary and major comments**

In this paper Sainburg et al are reviewing and describing the applications and usefulness of state-of-the-art machine learning technics for representing and analyzing vocal communication signals with no a priori assumptions on the informative bearing acoustic features of vocalizations. They use a recently developed dimensionality reduction method (UMAP), finding latent variables in that space. They show how projections of sounds used for communication in this latent space uncover the organization of various large datasets of vocalizations recorded in dozens of different species.

The authors are showing two major applications of UMAP: 1. on discretized vocal elements or in other words, on audio recordings already segmented into individual vocal elements such as syllables or phonemes; 2. on continuous bouts/sequence of vocalizations such as a bird or a mouse song. Finally, in the last sections, the authors quickly present various generative models of vocalizations based on neural networks that can be used to generate artificial vocalizations that respect the statistics of the vocalization repertoire, such as morphs between two existing real vocalizations. They show the results of behavioral and neural experiments in Starlings that aim to test the perceptual boundaries of this species with such morphs.

Through graphical representations of the projections, the authors are showing how known properties of segmented vocal elements, such as the presence of identity coding acoustic features, are nicely displayed in the UMAP latent space. They also demonstrate how UMAP combined with a hierarchical density-based clustering algorithm (HDBSCAN, developed by McInnes et al) can achieve unsupervised categorization of song syllables as well as, if not better than a trained human observer, in the sense that new sub-clusters, revealed by this unsupervised method, seem to indeed correspond to distinct syllable types as revealed by their different transition probability in the bird songs. The application of UMAP on non-segmented data, with projections of vocalization bouts as trajectories in the latent space rather than individual points for each individual vocalization element, is an interesting approach that is resolving one major problem in the analysis of animal communication that is often addressed using some more or less reliable heuristic: segmenting sequences in meaningful elements for the considered species. The continuous projection method seems a promising avenue for species in which the segmentation cannot be easily inferred from the structure of silence durations, behavioral observations or play-back experiments.

The originality of this article lays in the usage and application of very recent mathematical methods to bioacoustics. Along with all the material provided under Github, I can see how this article could be very useful for researchers working in animal communication to access and use computational methods that they might not have even think of otherwise and give ideas of new analysis. Moreover, the authors made a particular effort to apply these methods to a multitude of datasets, illustrating their power to an impressive diversity of questions in animal communication. However, the present lay-out of the paper set it half between a research/method/review paper. If I had to choose, I think I would stress more the method aspect to it, increasing the didactic aspect by giving more methods details (explaining/describing briefly UMAP and HDBSCAN, giving more metrics for quantifying the results of the projections, giving equations for the quantification metrics already used (e.g. Comparing algorithmic and hand-transcriptions section)). I would also add a short table of content at the beginning of the paper such that the reader can see the organization of the article and easily navigate it. As such it reads a little too much as a catalog of interesting, but “glimpsy” results.

Finally, the last section of the article 3.3.2, illustrating how generative models can be used in animal behavioral and neurophysiological studies, presents data that are referenced as being presented at a conference but seem unpublished. As such, there should be a section in the method of the paper regarding animal training, handling, fasting, surgeries, procedures, protocol etc…

**Line by line comments**

**Line 80-85**: I agree that an alternative approach to cherry-picked engineered features designed by humans such as pitch, duration is necessary, and data driven features are the most attractive. PCA can indeed be a good dimensionality reduction as long as it is applied to a representation of the data that is not already doing a strong compression. In the case of ref 24 (acoustic analysis of Humpback whales), PCA was applied on engineered features, measurements made on spectrograms of vocalizations. Such an approach is not resolving the problem pointed by authors line 76-79. PCA applied to a conservative feature space, such as well-designed spectrograms, has been implemented in previous studies, these might be better references (Mouterde et al, 2014, Plos One; Elie & Theunissen, Animal Cognition, 2015). I generally see DFA as a supervised classifier and while I understand the statement of the authors regarding its ability to extract, in a data-driven manner, the features that distinguish a certain categorization of the data, it all depends on what input is used for the DFA. If the parameters used are once again human-picked measurements as was done in the analysis of the Marmoset repertoire, then the problem of human-oriented results stay the same. Here again, a non-apriori feature space on which to perform the DFA could be the vocalization spectrograms as projected in the first dimensions of a PCA (see Elie and Theunissen, Animal Cognition, 2015).

**Line 103-104**

What do you mean by “high degree of control in real-world experiments”? What are real-world experiments?

**Legend Figure1:** maybe you want to prefer the word “segmented” to “discretized”
To what type of category do the categories referred to for each dataset correspond? Acoustic categories made by the authors? Meaning categories made by inference from behavioral observations? Identity of the vocalizer?

**Line 157-175:** Which metrics can be derived from the latent projections to quantify within and compare across species the potential for identity coding?

Here you selected vocalization types that we know by behavioral observation/playback experiments are used for individual recognition, would you have found that structure if you had been given the repertoire without any indication?

**Figure 5:** I’m curious about the individual contributions of UMAP and HDBSCAN to the clustering “performance” obtained with the combination of both methods. Did the author tested HDBSCAN/spectrogram? Can the contribution of each be quantified?

**Figure 8:** First and second row of the figure are said to be the same but color coded by time (upper row) or hand-labels (lower row), however there seem to be missing points in the second row, why is that? Can a mention of this difference be explained in the legend of the figure?

Also, the legend of what I guess being the hand-labels colors in M and N is missing.

Finally, it could be useful to describe the colorspace projections of the UMAP projections in the methods, instead of having to dive into the code. Would help to understand what we’re looking at in terms of color scheme.

**Line 674-681:** Comparing algorithmic and hand-transcriptions section: The equations of the various metrics should be given.

**Section 3.1.4:** I understand the power of UMAP and HDBSCAN for automatically inferring the syllables categories from the data, but I don’t see the extra degree of abstraction that one gets for the sequential organization of syllables with the projection in latent space.

**Figure 15 E and F** how is the similarity measure calculated?

**Minors comments:**

**Line104 “control”** written twice

**Line 124: “Supplementary”**  should be supress

**Line 197: To compare**

**Figure3:** legend miss-ordered

**Line 235:** sequences **of** a few

**Line 253:** you probably want to refer to figure 7 D **K**?

**Line 318:** a “strong hue”? a similar hue ?

**Line 338:** reference to figures inversed (Finch -> 8; starlings -> 9)

**Line 343:** Wouldn’t 8C instead of 8I be the good comparison here, in terms of color labelling?

**Legend figure 10**: the first and second **USV** dimensions are plotted as color dimensions -> the first and second UMAP dimensions are plotted as color dimensions?

**Line 417**: remove “In the”

**Line 448**: missing closing parenthesis

**Line 448**: add the abbreviation here to define MDS-AE

**Legend figure 12**: MD-AE - > MDS-AE; LSTM is not defined, Various D_x_ variables are not defined.

**Legend figure 16**: a word is missing in “The algorithm dynamically a noise threshold based upon…. »
