# Supplementary material for: Finding, visualizing, and quantifying latent structure across diverse animal vocal repertoires
Source: PLoS Comput Biol. 2020 Oct 15;16(10):e1008228. doi: 10.1371/journal.pcbi.1008228 (PMC7591061; doi:10.1371/journal.pcbi.1008228)
Supplement: Attachment — Submitted filename: Sainburg et al Review.docx [file pcbi.1008228.s010.docx]

Sainburg et al apply UMAP to bird song. This allows bird song syllables - time series of various lengths, often with considerable spectral complexity - to be projected into a small number of dimensions. They apply this idea to syllables and sequences of syllables. In addition, they use latent space models (this time with neural networks) to generate syllables from a data-set. In my opinion, this is the more novel and interesting part of the paper. Bird song is a major model for the neuroscience of learning, cultural evolution in non-human animals, and communication. Methods to improve our ability to computationally compare learned songs are therefore extremely valuable.

I feel that this paper represents an interesting advancement of this topic. I am not convinced by all the authors arguments about how their approach removes assumptions (see below), but nevertheless it is a valuable step in the right direction for the field.

General comments:

I was unfamiliar with UMAP before reviewing this manuscript, and have read through the documentation at this website: https://umap-learn.readthedocs.io/. This has led to some questions that I was unsure of from the manuscript:

(1) In the Methods, I found that there were not enough details in the Projections section. According to the documentation, there are quite a few settings to be made for UMAP (learning rate etc).

(2) How was the data entered into UMAP? I understand that the data for a particular syllable consisted of the power values from the cells of the 32 x 32 transformation of the spectrogram. Is it the case that each of these values consisted of a separate “dimension” of the data entered into UMAP?

(3) If so, then would it be correct to argue that the comparison method underlying this paper is - ultimately - a form of spectrogram cross-correlation? The more similar the power values of cells in the two spectrograms, the closer they will be projected together into the low-dimensional space?

This leads me to my main critique of the manuscript, as I understand it. The original computational study comparing bird song was the spectrogram cross-correlation paper by Clark et al 1987. In that paper, spectrographic cross correlation was used to generate a dissimilarity matrix between the units of song compared. Then principle coordinates was used to project this into low-dimensional space. If I understand your work correctly, you have essentially updated this approach by using UMAP instead of principle coordinates. This provides some advantages (below), but the ultimate measure dissimilarity is rather similar to spectrogram cross-correlation. Is it not true that you could have used any other measure of song comparison (e.g. Sound Analysis Pro, DTW, parametric etc) as the input to UMAP (especially because according to the docs, UMAP will accept N x N dissimilarity matrices…)?

This is relevant because cross-correlational methods do make quite strong assumptions about what constitutes similarity between acoustic signals. And therefore, if I have understood your method correctly, I disagree with your characterisation of your method using unusually few assumptions of the data. For example, your methods make assumptions about the relative weighting of frequency versus temporal shifts in that once a change has been made large enough to shift energy into a different cell in either domain, then similarity will rapidly drop-off. For another, you chose to pad syllables at the end to make all spectrograms the same length - this would give different results from padding at the beginning. Cross-correlational methods also have other problems: they tend to be “brittle” in that as soon as there is no overlap between spectrograms, then similarity will drop to zero, and it can be rather hard to predict when this happens. From what I can tell, the same would be true in this case, unless UMAP is able to correct for it. So for example, imagine that two chevron-shaped notes were sampled, of identical ‘shape’ on the spectrogram, but separated by 500Hz in absolute frequency. I would imagine that no similarity would be found between them using this new approach… unless enough additional similar notes are also sampled which are intermediate in structure (in which case the nearest-neighbour approach used by UMAP may reconstruct the correct relationship between the two original notes.

(4) The UMAP documentation suggests that the algorithm is primarily to be used for visualisation, and even its authors say that its use for clustering is “somewhat controversial and should be attempted with care”. There is no discussion of this potential limitation in the manuscript. One issue is that UMAP may tend to generate artificial clusters.

(5) One issue with your method is that, like other “whole spectrogram” methods, it may be particularly susceptible to background noise or reverberation. Did you test this? In Figure 1, it seemed to play a role in some judgements.

(6) In Figure 1, my impression was that the analysis did not perform well in cases where there were a large number of categories, but with some categorical structure. For example, in the case of swamp sparrows, the note examples in the top left has clear resemblance to the note examples in the bottom-right and in the bottom-left (in all cases, relatively long notes that descend in frequency at an increasing rate), despite these being in very different positions on the projection. There was a similar pattern with the Cassin’s vireo. The two note examples in the top left show a small chevron followed by a more prominent upsweep in frequency. That same pattern is found in another note towards the bottom right - placed in a cluster that is very far away in the projection. In the case of starlings, there are several cases where highly harmonic chevron shapes predominate, yet these are projected to very different places. These results seemed to go very clearly against what I would predict from other analyses of similarity, or from subjective human judgment. What validation of these results can you provide? Is it possible that this method will effectively separate different note type categories, but still fail to reflect more global patterns of structure?

(7) Overall, I think the paper would benefit from clearer, quantitative efforts to compare the output of this analysis method with previous methods.

(8) Section 3.3 seems to really be a different paper. The links between 3.1/2 and 3.3 are rather tenuous - the methods involved are really quite different. While I think both are interesting, I wonder whether a more focused and useful result could be generated by splitting this into two papers.

Specific Comments

Line 66-79 - while I agree with the thrust of this paragraph, is it not also the case that spectrograms are a particular and not particularly perceptually valid representation of the data (they are not “the data” as you say in the next paragraph). And its not immediately obvious that all features would be accessible to your method. For example, relative pitch or Wiener entropy are quite ‘perceptible’ to us (‘melody’ and ’spectral noisiness’) but I do not see how your algorithm could detect changes in either.

Lines 80-81 - there’s an assumption here that the structure of the data (produced vocalisations) will reflect animals’ perception. That’s not necessarily the case - there might also be, for example, physical limitations on sound production which mean that the structure of the data deviates from how animals perceive it. In the case of highly clustered data (like learned bird song), there’s an additional question of how much data you would need to collect before it provided the information needed to shape the manifold (for example: does the vocal repertoire of one zebra finch provide enough information to learn the structure of zebra finch vocalisations?). I don’t disagree with your argument here, but believe that its own assumptions and limitations need to be fleshed out a little more fully.

Lines 85-86 - this seems like unsupported assertion. (Or maybe I don’t understand better than what?)

Lines 95-96 - I disagree with this assertion. I think that your method makes as many assumptions as any other method.

3.1.1 - It is not surprising that species differ in their clustering tendency when the data-sets are so different: some are from just one individual while others are from very many. I didn’t really understand the comment in the legend of Fig.1 about how the choice was made to use one or many individuals. In the case of swamp sparrows, individuals certainly have distinctive song repertoires (as much as zebra finches, for example). I think, for example, that had you sampled syllables from a population of zebra finches, you would have found little clear clustering.

I think this point applies to the comparisons made between birds and mammals and between birds and human speech (3.1.3) too.

Lines 154-156 - these claims might also apply to naive cross-correlation methods too.

Line 189 - In this case, the classification rate of the two whale species does not seem particularly high, and you refer to other work used to identify species. Can you compare your classification success with other methods?

Line 195 - I don’t think “dialects” is the correct term here - “regiolects” might be more accurate. In addition, the categorical perception in swamp sparrows is found within populations, not between populations.

Line 197 - typo compared -> compare

Fig.3C and commentary - Unfortunately, the projection of swamp sparrow notes really doesn’t match with any structure found in previous studies, so this result is hard to evaluate (also - you can get a clearer representation with a similar conclusion simply by plotting peak frequency change versus note length for these very simple notes). The good thing about swamp sparrow notes is that they are simple enough to capture even with just a couple of features, which might allow you to test the performance of your method. One thing I would like to know is whether the buzzy, frequency modulated notes that swamp sparrows sometimes use show up as a separate cluster or not.

Figure 3 - A and B are labeled the wrong way around in the legend.

Table 1: Finch should be labeled zebra finch.

Appendix: in the case of swamp sparrow notes, there seem to be broad-band artefacts at the end of most notes.

3.1.4/3.2 Lachlan et al. 2013 (Current Biology) also used a method to abstract sequential structure from the structure of the data - looking for categories that maximised redundancy. It would be interesting to apply this method to your UMAP clustered data. This method was developed for data that was not clearly clustered…

392: again, I really don’t think you can argue that your method is less biased than other studies.

Categorical perception experiment - categorical perception relies on stimuli evenly drawn from a continuous physical dimension being warped in perception. I am not completely sure you can claim that your latent representations are evenly reflecting a continuous dimension. The very fact that these methods allow non-linear dimension reduction is surely an issue here?

3.3.1 - I’m not entirely sure what the conclusion to draw from this analysis should be.

Fig. 14 - the figure legend doesn’t explain what d is… and I couldn’t make sense of the labels above the figures, in light of the figure legend below.

3.3.2 - Playing devil’s advocate a bit, but: in 14A and 15B, as you go from one syllable to another, it almost looks like a case of masking. In the top-left of 14A, for example, the “volume” of the top-left original is at maximum, and the “volume” of the top-right original is at 0. As you move along the top row towards the top-right, the “volume” of the top-right original is gradually turned up, and that of the top-left original is turned down. In the middle, both original stimuli are played at equal volume.

If that is all that is happening, perhaps the subsequent behavioural/neurobiological results are fairly understandable?

Methods:

I would appreciate a fuller and clearer explanation of how choices were made to log transform frequencies or not; and how spectrogram data were entered into the UMAP algorithm. It would be good to know how sensitive the results were to the choice of 32 x 32 bins for the spectrogram. How would 16 x16 or 64 x 64 differ?

The details for the neural networks seem quite sparse. This reinforces my feeling that 3.3 belongs as a separate paper.
